# Corresponding Functional Dynamics across the Hsp90 Chaperone Family: Insights from a Multiscale Analysis of MD Simulations

**DOI:** 10.1371/journal.pcbi.1002433

**Published:** 2012-03-22

**Authors:** Giulia Morra, Raffaello Potestio, Cristian Micheletti, Giorgio Colombo

**Affiliations:** 1 Istituto di Chimica del Riconoscimento Molecolare, CNR, Milano, Italy; 2 Scuola Internazionale Superiore di Studi Avanzati (SISSA) and CNR-IOM Democritos, Trieste, Italy; 3 Max Planck Institute for Polymer Research, Mainz, Germany; UNC Charlotte, United States of America

## Abstract

Understanding how local protein modifications, such as binding small-molecule ligands, can trigger and regulate large-scale motions of large protein domains is a major open issue in molecular biology. We address various aspects of this problem by analyzing and comparing atomistic simulations of Hsp90 family representatives for which crystal structures of the full length protein are available: mammalian Grp94, yeast Hsp90 and *E.coli* HtpG. These chaperones are studied in complex with the natural ligands ATP, ADP and in the Apo state. Common key aspects of their functional dynamics are elucidated with a novel multi-scale comparison of their internal dynamics. Starting from the atomic resolution investigation of internal fluctuations and geometric strain patterns, a novel analysis of domain dynamics is developed. The results reveal that the ligand-dependent structural modulations mostly consist of relative rigid-like movements of a limited number of quasi-rigid domains, shared by the three proteins. Two common primary hinges for such movements are identified. The first hinge, whose functional role has been demonstrated by several experimental approaches, is located at the boundary between the N-terminal and Middle-domains. The second hinge is located at the end of a three-helix bundle in the Middle-domain and unfolds/unpacks going from the ATP- to the ADP-state. This latter site could represent a promising novel druggable allosteric site common to all chaperones.

## Author Summary

Understanding the connections between structure, binding, dynamics and function in proteins is one of the most fascinating problems in biology and is actively investigated experimentally and computationally. In the latter context, significant advancements are possible by exposing the causal link between the fine atomic-scale protein-ligand interactions and the large-scale protein motions. One ideal avenue to explore this relationship is given by proteins of the Hsp90 chaperones family. Their dynamics is regulated by ATP binding and hydrolysis, which activates the onset of large-scale, functional conformational changes. Herein, we concentrated on three homologs with markedly different structural organization—mammalian Grp94, yeast Hsp90 and prokaryotic HtpG—and developed a novel computational multiscale approach to detect and characterize the salient traits of the functionally-oriented internal dynamics of the three chaperones. The comparative analysis, which exploits a novel highly simplified, yet viable, description of the protein internal dynamics, highlights fundamental mechanical aspects that preside the ligand-dependent conformational arrangements in *all* chaperones. For the three molecules, two corresponding regions are singled out as ligand-susceptible hinges for the large-scale internal motion. On the basis of this and other evidence it is suggested that these regions represent functionally relevant druggable substructures in the discovery of novel allosteric modulators.

## Introduction

In recent years, experimental and theoretical studies have increasingly focused on promiscuous proteins [1], [2]. These are biomolecules that bind and process different ligands and include hub proteins with central roles at the crossroads of distinct cellular pathways [1], [3], [4]. To satisfy the diversity of their functional tasks and recognize structurally different partners, these proteins must visit different conformational states. The associated structural rearrangements are in many cases controlled by allosteric mechanisms [5], [6]. In particular, ligand binding or local chemical modifications can trigger large-scale conformational changes impacting on the recognition, binding and reactivity properties at distal sites [7].

One notable instance is represented by the 90 kDa heat shock (Hsp90) family of proteins [8]. These homodimeric multidomain molecular chaperones are highly conserved from bacteria to eukaryotes and oversee the correct folding and conformational maturation of several client proteins [9], [10], [11], [12], [13]. Crystal structures of full-length Hsp90 constructs have been obtained for yeast Hsp90 [14], the mammalian endoplasmatic reticulum (ER) paralog Grp94 [15], [16] and the bacterial homolog HtpG [17].

**Figure 1:**
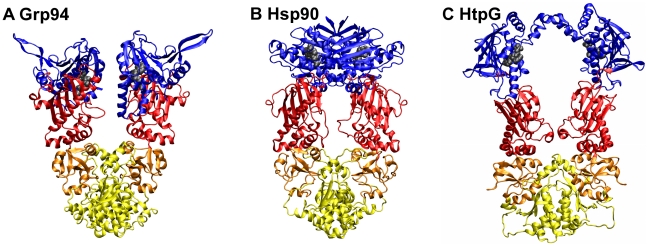
Starting structures used for the MD simulations. **A**, canine ATP-bound Grp94 structure, PDB entry 2O1U, with ATP lid and missing loops modeled as described in Materials and Methods. **B**, yeast ATP-bound Hsp90 structure, PDB entry 2CG9, with charged linker and disordered loops modeled as described in [35]. **C** HtpG structure, PDB entry 2IOP.pdb, corresponding to the ADP-bound form resolved in [17]. The structural domains are colored as follows: blue, N-terminal domains; Red, M-large domains; Orange, M-small domains; Yellow, C-terminal domains. The domains span the following sequence intervals: Grp94 (NTD: 85–285; M-large: 330–511; M-small: 512–602; CTD: 603–749); Hsp90 (NTD: 2–215; M-large: 264–426; M-small: 427–526; CTD: 527–676); HtpG (NTD: 1–215; M-large: 216–400; M-small: 401–490; CTD: 491–624).

HtpG, Hsp90 and Grp94 have a mutual sequence identity of about 45% which reflects in the good structural alignability of their individual domains. However, the preferential relative orientation of the domains varies significantly depending on the reference organism and cellular compartment (**Figure 1**) [17], [18], [19], [20], [21], [22], [23]. Consequently, while the structural RMSD of optimally aligned individual domains of these homologues is typically of the order of 2 Å, the alignment of the whole crystal structures yields an RMSD of about 7.75 Å.

In general, Hsp90 functions depend on ATP binding and hydrolysis, and play a pivotal role for controlling multiple signaling networks associated with cell viability [24], [25], [26], [27], [28]. At the same time, its pathological activity, associated to overexpression and up-regulation, is a key molecular factor in favoring and maintaining the disease states in several cancers and neurodegenerative diseases. A prominent strategy for the development of chemotherapeutics is indeed focused on the inhibition of Hsp90 in tumor cells, particularly by targeting the N-terminal ATP-binding site [29]. The full exploitation of such drug-leads in the clinical setting is however hampered by dosage, tolerance and solubility problems [29], [30], [31]. The discovery and exploitation of alternative functional, druggable sites may generate novel opportunities for drug development, overcoming the limits associated with the classical approach towards Hsp90 inhibition [32], [33], [34].

In a recent atomistic molecular dynamics (MD) study of Hsp90 [35], some of us have used inter-amino acid fluctuations to propose a site, distant from the ATP-binding pocket, against which several inhibiting ligands were successfully screened. These ligands showed good anticancer activities in experimental tests, along with the capability to block physical interactions between Hsp90 and client and co-chaperone proteins [36].

The above-mentioned studies [35], [36] demonstrate the feasibility of discovering novel inhibitors of these chaperones by suitable analysis of their detailed functionally-oriented internal dynamics.

This approach could have an even greater potential if it could exploit or target the functional dynamical aspects shared by the homologue chaperones despite their different overall structure. Such mechanistic properties, being at the intersection of the functional dynamics of different proteins, would expectedly be crucial for the molecules' activity and their characterization could help the discovery of potent inhibitors.

To make progress in this appealing avenue, which complements previous approaches where chaperones' differences rather than similarities were analyzed [16], it is necessary to compare the internal dynamics of structurally-different proteins. Presently available tools, such as the dynamics-based alignment [37], [38], [39], [40], [41], [42], [43], allow for carrying out this challenging task only for proteins whose structural fluctuations are sufficiently small to be viably projected onto few essential dynamical modes [44], [45].

Here we take a first step towards formulating a dynamics-based, multiscale comparative scheme that is more general and hence applicable to structurally-different proteins whose internal dynamics has a very large amplitude. Specifically, the internal dynamics of different proteins is compared from the fine level of inter-residue fluctuation to the much larger one of domain motion. The latter is captured with a minimalistic quasi-rigid domain description which allows for a transparent and intuitive dynamics-based comparison.

The strategy is applied to the atomistic simulations of the three Hsp90 chaperones in the ATP-, ADP- and Apo states, covering a total time span of 900 ns. It is found that two regions are arguably involved in modulating the well-known ligand-dependent large-scale motion of the three chaperones [17], [18], [19], [20], [21], [22], [23], [46], [47], [48]. One of them corresponds to a previously identified functional site at the interface between the N-terminal and Middle-domains [20], [49], [50], [51]. The second site is located at the interface between the large and small sub-portions of the protomeric Middle domain (called M-large and M-small, respectively) (**Figure 1**). To the best of our knowledge, the functional role of this site was not investigated before and, based on the evidence gathered here, could represent a promising novel target for small molecule inhibitors of all the considered chaperones.

## Results

### Ligand-dependent conformations of Hsp90, Grp94 and HtpG

Proteins of the Hsp90 family are homodimers. Each protomer consists of three structural domains: an N-terminal regulatory Domain (NTD), responsible for ATP binding, a Middle Domain (M-domain), which completes the ATPase site necessary for ATP hydrolysis and binds client proteins, and a C-terminal Domain (CTD) that is involved in dimerization [14], [16], [17], [21], [52]. The currently available repertoire of Hsp90 homologues from different organisms consists of three crystal structures. They correspond to the mammalian Grp94, the yeast Hsp90, and the procaryotic HtpG. Reference structures for the three chaperones are shown in **Figure 1**. The same figure highlights their standard partitioning into four structural protomeric domains: the N-terminal (NTD), Middle large (M-large), Middle small (M-small) and C-terminal (CTD) domains. The protein segments covered by each of the three domains are provided in the figure caption.

It is seen that the full-length construct of Grp94, in complex with either ATP or ADP, is a semi-open, V-shaped dimer held together by a twisted C-terminal interface [16]. An analogous C-term interface is found in the ATP-bound Hsp90 where, in addition, the N-terminal domains are in contact too [14] because of the swapped α-helix1 and β-sheet1. Finally, in ADP-bound HtpG, the two protomers are extended and nearly parallel. In this structure, the contacting C-terminal domains are not twisted (See Supporting Information **Figure S1**), and the rest of the dimeric interface involves a few contacts, such as α-helix2 and α-helix3 from the NTD, one of the edges of the M-large subdomain and the tips of the ATP lids.

### MD simulations

The available crystal structures of full-length Grp94, Hsp90 and HtpG were used as starting points for extensive atomistic MD simulations, see Materials and Methods. As a general caveat, one should consider that, compared to the time-scale accessible in experiments, those accessible in our simulations are much shorter and are typically in the order of 100 ns.

Specifically, the MD trajectories of yeast Hsp90 were obtained by extending to 100 ns those carried out by some of us in a previous study [35]. Within the limits of MD sampling, the extended trajectories confirmed that the N-terminal interface remains tightly closed in the compact ATP-bound structure. In the presence of ADP it instead becomes more exposed to solvent with local rearrangements of the interface helices. This ligand dependent behavior is reflected by the C-terminal interface, which has a larger solvent accessible surface area in the ADP-state compared to the ATP-state, see **Figure S2**. Such difference is not expected *a priori* given the rather closed character of the dimeric conformation of nucleotide-bound Hsp90.

The behavior of the Grp94 and HtpG trajectories is much richer and it indicates that the relative positioning of the N-terminal and Middle domains is strongly affected by the type of bound ligand, as detailed below. The final structures of all simulations are provided as supplementary information files **Text S1, S2, S3, S4, S5, S6**.

#### ATP-state

**Figure 2:**
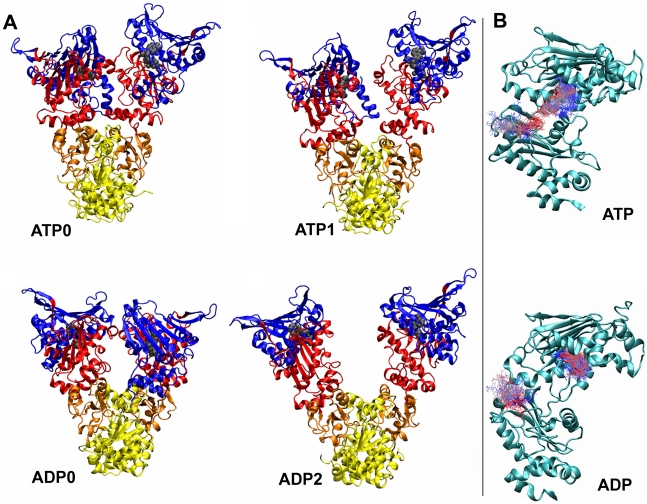
Representative structures of Grp94. **A**, Labels ATP0 and ATP1 show the representative structures of the two most populated cluster in the Grp94-ATP simulation. Labels ADP0 and ADP2 show the representative structures of the first and third most populated cluster in the Grp94-ADP simulation. The second cluster (not shown) represents the intermediate between ADP0 and ADP2, characterized by a slight rotation of the NTDs and an initial opening of the M-domains. **B**, trajectory snapshots showing the time evolution of the interaction between the nucleotide and Arg448 in the ATP simulation (top) and in the ADP simulation (bottom). Snapshots are color coded according to time evolution: from blue (0 ns) to red (100 ns).

In the ATP-state of Grp94, the initial proximity of the N-terminal and Middle domains is maintained mostly by the electrostatic attraction of the nucleotide and the oppositely-charged Lys residues (364,404,405) and by the interaction of hydrophobic residues such as Val416, Phe417 and Ile418 (M-domain) with Leu117 (N-term domain). In the course of the MD evolution the relative orientation of the N-terminal and Middle domains is also preserved. The ATP-lid, which completes the nucleotide binding site in the NTD, populates mainly the starting “open” state and, towards the end of the trajectory, the “extended open” state. The latter lid conformation is induced as the NTD of one protomer approaches the N- and M-domain of the opposite protomer (**Figure 2**). Arg448 and Glu103 (corresponding to Arg380, Glu33 in Hsp90), which are required for catalytic activity, are coordinated with the ATP nucleotide (See **Figures 2** **, S3**).

**Figure 3:**
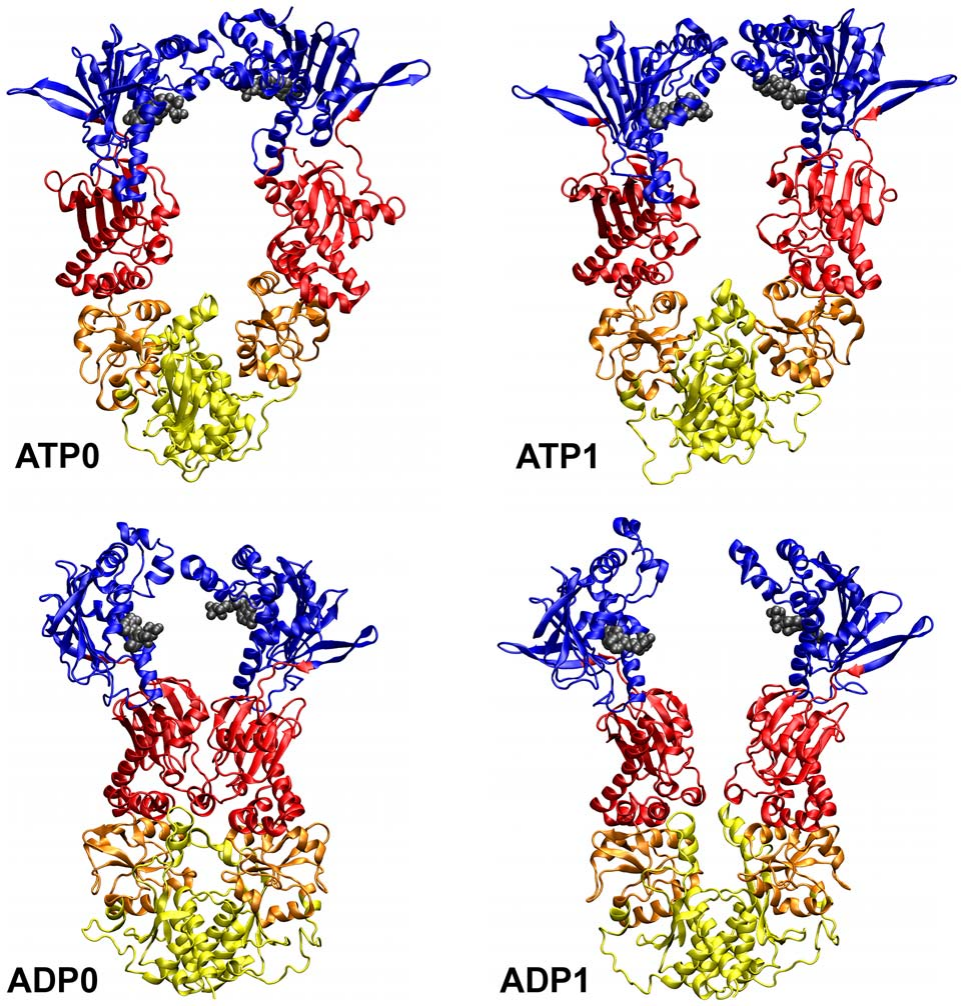
Representative structures of HtpG. Labels ATP0 and ATP1 show the representative structures of the two most populated clusters in the HtpG-ATP simulation. Labels ADP0 and ADP1 show the representative structures of the two most populated clusters in the HtpG-ADP simulation.

For the ATP-bound HtpG the number of contacts between the NTDs, which initially involve only the tips of the ATP-lids (residues 106–115), increases during the MD evolution. The tightening of the NTDs interface is favored by the relative rotation of the two protomers which, in addition, causes an increase of the inter-protomer distance between the Middle and C-terminal domains and exposes residues 11,12,15 in α-helix1 to the interaction with the ATP lid of the partner protomer (**Figure 3**). As a consequence, the ATP-bound structures have an overall less compact organization than the starting (ADP-bound) crystal structure, consistently with experimental indications [53].

#### ADP-state

Significant conformational changes occur in the MD trajectory started from the ADP-bound structures. In Grp94 the ATP-lid, which was initially modeled in the “open” state (see Material and Methods), undergoes a conformational change that populates mainly the “extended open” state [16] (**Figures S4**). Moreover, the N-terminal domain is canted with respect to the Middle one. This rearrangement is promoted by the fact that the electrostatic attraction of Lys364, Lys404 and Lys405 (in the Middle domain) with the nucleotide bound in the NTD is weaker for ADP compared to ATP. Because of the increased separation of the N-terminal and M-domains (**Figure S3, S5**), the catalytic loop is less constrained and Arg448, Glu103 lose the preferential orientation towards the nucleotide-binding site. The effect is illustrated by the ADP induced variation of the Lys-nucleotide minimum distances reported in **Table 1** for all the three proteins. For Hsp90, we point out that the two, initially contacting N-terminal domains are still partially in contact after extending the MD evolution to 200 ns (**Figure S6**). Although the time-span covered by the present simulations is, as expected, not sufficient to reach a state with fully-detached NTDs, our analysis highlights specific interactions responsible for the onset of the motions inducing N-terminal dissociation.

**Table 1:** Final and Average Distances between ATP or ADP and residues important for binding and catalysis in Grp94, Hsp90 and HtpG.

Fin/Avg. dist (nm)	ATP Grp94			ADP Grp94		
	Fin1	Fin2	Avg	Fin1	Fin2	Avg
**Lys364**	0.61	0.39	0.57	1.46	0.30	0.98
**Lys404**	1.38	0.27	0.80	2.24	0.84	1.57
**Lys405**	1.09	0.77	0.91	2.06	0.23	1.27
**Arg448**	0.42	1.32	1.34	2.05	0.64	1.76

	ATP Hsp90			ADP Hsp90		
	Fin1	Fin2	Avg	Fin1	Fin2	Avg
**Lys294**	2.46	2.31	2.20	2.97	2.69	3.23
**Lys337**	2.42	1.88	2.24	3.07	2.45	2.73
**Lys338**	1.68	2.49	2.51	2.71	2.66	2.73
**Arg380**	0.20	0.24	0.27	0.43	0.42	0.52

	ATP HtpG			ADP HtpG		
	Fin1	Fin2	Avg	Fin1	Fin2	Avg
**Lys251**	3.64	3.63	3.56	3.81	4.03	3.85
**Lys294**	3.37	3.52	3.25	3.78	3.78	3.60
**His295**	3.28	3.50	3.34	3.72	3.71	3.62
**Arg336**	2.19	2.13	2.12	2.65	2.65	2.55

The distance is calculated between the closest pair of atoms at every timestep.

Consistently with previous observations for Hsp90, the C-terminal solvent accessible surface in the ATP-state is smaller than in the ADP-bound one because the mobility of the two C-terminal loops is restricted by the nearby M-small subdomain (**Figure S1, S2** and **Figure S5**).

In the ADP-state of HtpG, an opening of the clamp-like structure resulting from the partial NTDs association is observed. Such conformational change is induced by the increased mobility of the domains due to the loosening of the protein electrostatic interactions with ADP compared to ATP. This, in turn, is followed by the approach of the two M-domains and results in a stable interaction surface of the protomers (See **Figure 3**).

Finally, the effects of the ligands on HtpG C-terminal interfaces are consistent with that observed for the eukaryotic cases, despite the different structural organization of the terminal domains in the prokaryotic protein. Namely, ADP induces an increase of the C-terminal solvent accessible surface with respect to ATP (**Figure S1 and Figure S2**).

#### Apo-state

Interestingly, in the Apo-state of Grp94, the ATP-lid is very flexible and locally unfolded and visits both the “open” and the “extended open” conformations, indicating that no specific conformational preference exists in the absence of ligand. The N-terminal domain can move significantly with respect to the Middle one in the absence of the nucleotide (**Figure S5**). In Hsp90 this behavior is not observed due to the extremely long simulation time it would take to unwind the tightly packed N-terminal interface. Analogous considerations also apply to the Apo structure of HtpG, where the initially-contacting NTDs presumably restrict the conformational space visited in the simulation timescale. In fact, as noted before, because of the necessarily limited duration of atomistic MD simulations, the starting conformations of the models may have a significant impact on conformational sampling of these complex molecular assemblies. Notwithstanding these expected difficulties, it is remarkable that sizeable structural rearrangements are observed across these trajectories and therefore provide valuable indications into the innate inter-domain motions that the chaperones can sustain in thermal equilibrium.

The above results suggest that the three chaperones share the following aspects of the ligand-dependent conformational rearrangements. In the ATP-state, the two NTDs tend to approach and clasp favoring a closed dimeric conformation. A more open chaperone conformation ensues upon substituting ATP with ADP. This follows from the reduction of the electrostatic attraction between the N-terminal and Middle domains. As a consequence, the Grp94 chaperone populates a conformation akin to the “open-compact” structure characterized by Agard and coworkers in the presence of ADP [17], [19] (See **Figure 2**).

### Ligand-dependent fluctuations of aminoacid pairwise distances

The presence and type of bound ligands profoundly affect the internal dynamics of Grp94, Hsp90 and HtpG both locally and globally [14], [16], [17], [23], [54], [55], [56], [57], [58], [59], [60].

**Figure 4:**
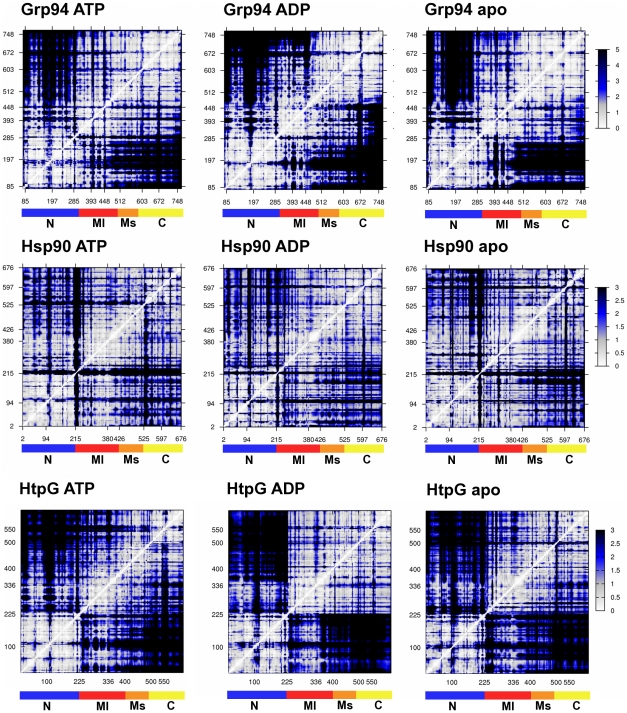
Distance fluctuation matrices for Grp94, Hsp90 and HtpG protomers in all different ligand states. The magnitude of pairwise distance fluctuations is color coded from white (small fluctuations) to black (large fluctuations). The different absolute scale of pairwise fluctuations between Grp94, Hsp90 and HtpG reflects the different internal mobility of the three molecules.

As a first step of the analysis of the short- and long-ranged ligand-dependent structural changes, we computed the fluctuations of pairwise amino acid distances in the MD trajectories of the three proteins (see Materials and Methods). The pairwise mean-square distance fluctuations are shown in the color-coded matrices of **Figure 4**.

From an overall point of view, all matrices exhibit a similar block character, which reflects the alternation of regions with small and large fluctuations of inter-residue distances [42], [43]. As discussed hereafter, the correspondence extends to finer details of the matrices which point to detailed ligand-dependent modulations of the protomeric structures in the three chaperones.

We first compare the matrices relative to Grp94 in the ATP- and ADP-state.

In **Figure 4**, it is readily seen that the fluctuations of the distances of segments 285–448 and 672–748, respectively in the M-large and C-terminal domain, are larger in the ADP-state compared to the ATP one, indicating an increased relative motion of the two domains.

More in detail, the change of ligand increases the mobility of the catalytically crucial residue Glu103 (on average 0.1 Å^2^ with ATP and 0.5 Å^2^ with ADP) and of segment 448–510, the principal helix of the Middle domain, with respect to the M-small and C-terminal domains, as indicated by higher values of distance fluctuation (the average distance fluctuation between the stretch 448–510 and residues over 510 is 1.7 Å^2^ with ATP and 3.2 Å^2^ with ADP). This segment includes part of a cluster of aromatic residues at the end of the H4–H6 three-helix bundle (Phe484, Phe488 and Phe432), located at the boundary between the M-large and M-small structural subdomains [14], [22]. This group of residues is optimally packed in the starting structure and remains so in the presence of ATP. By contrast, it undergoes a progressive local unfolding/unpacking in the presence of ADP (**Figure S7**). Interestingly, this local unfolding shortly precedes the opening transition of the dimeric clamp (see Supporting Information **Figure S8**).

Local mobility of the flexible linker connecting the M-small and C-terminal domain, around residue 603, increases in the presence of ATP with respect to ADP: the average distance fluctuations within segment 590–610 nearly double going from ADP (0.5 Å^2^ on average) to ATP (1 Å^2^ on average).

The above mentioned effects are also seen in the Hsp90 and HtpG matrices (the former only having a smaller average amplitude of distance fluctuations because of the tighter dimeric structural organization). In particular, changing the nucleotide from ATP to ADP results in the unpacking/unfolding of the end of the H4–H6 three-helix bundle in the M-domain, corresponding to residues 427–432 in Hsp90 and residues 360–390 in HtpG. In addition, the increased fluctuation amplitude of the linker connecting the M-small domain and the C-terminal domain (around residue 525 in Hsp90 and 500 in HtpG) is also observed.

### Ligand-dependent large-scale motions: mechanical hotspots

Additional insight into mechanistic functional aspects of Grp94, Hsp90 and HtpG is obtained by considering their ligand-dependent geometric strain, that is the ligand-dependent local deformations of the residues' contact networks (see Materials and Methods) [41].

**Figure 5:**
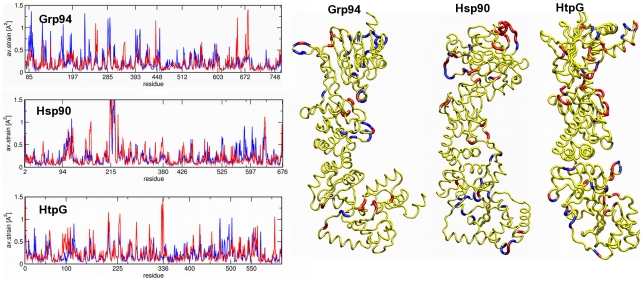
Geometrical strain analysis. Left panel, the residue based profile of average strain calculated over the time interval 20–100 ns for the three proteins in ATP-bound state (blue line) and in the ADP-bound state (red line). Right panel, protein monomers with average strain maxima shown in blue (ATP case) and red (ADP case).

**Figure 5** portrays the geometric strain for Grp94. The figure illustrates the protomer- and time-averaged strain profiles for the ADP- and ATP-states. A typical time evolution of the strain is shown in **Figure S9**.

It is seen that a recurrent buildup/release of geometric strain occurs in regions that either are directly involved in chaperone's functional activity or correspond to exposed loops. Restricting considerations to the first type of regions, one has that the strain hotspots in the ADP state correspond to (i) the linker connecting the N- with the M- interface (residues 340–343), (ii) the catalytic loop around Arg448, (iii) the interface region separating the M- and C-terminal domains (residues 573–575) and finally (iv) the C-terminal loops (residues 652–682) in the vicinity of a previously identified C-terminal binding pocket for allosteric inhibitors [36]. In the ATP state, besides the strain buildup in correspondence of the above regions, a diffuse strain pattern is established around the N-terminal ATP-lid and the linker connecting Middle and C-domain around residue 603.

These results have a parallel in the Hsp90 strain pattern, also shown in **Figure 5**. In fact, in the ADP state the strain hotspots correspond to: (i) the N-terminal/Middle domain interface (residues 185–220), (ii) the catalytic loop (residues 375–383), (iii) the M-C interface (residues 484–495), and (iv) the C-terminal residues near the allosteric binding region (residues 640–650). In the ATP state, high strain regions are limited to the ATP-lid, the linker connecting the Middle and C-terminal domains (centered on residue 525) and the core of the C-terminal domain (residues 555–565 and 570–575).

Analogous results are found for HtpG too. As seen in **Figure 5**, its strain hotspots in the ADP state are: (i) the N-M interface (residues 180–190), (ii) the catalytic loop (around residue 336), (iii) the interface between Middle and C-terminal domain (residues 450–490) and (iv) the C-terminal loops (residues around 550). In the ATP-state high average strain is found at the N-terminal domain, involving the initial secondary structure motifs, at the ATP lid and also at a rather extended region comprising the linker between Middle domain and C-terminal domain (residues 475–500).

In *all* systems, the catalytic Arginine (448 in Grp94, 380 in Hsp90 and 336 in HtpG) shows an increase of conformational strain in the presence of ADP with respect to ATP. The increased strain reflects a reduced correlation of the motion of the catalytic loop and the bound nucleotide. The inspection of the MD trajectories indicates that ADP-dependent increased fluctuation of the catalytic loop reverberates on the motion of the proximal Middle domain helix, which triggers the local rearrangement at the Middle domain three-helix bundle H4–H6 (see **Figure S7**). This suggests that the catalytic Arginine acts as a sensor for the nucleotide state. Indeed, in the presence of ADP, the increased local mobility of the catalytic Arginine releases initial structural/electrostatic constraints on the principal helix (H3) of the Middle domain, triggering the motion of this helix as a rigid body. Finally, no ligand-specific strain signal is evident for the highly-charged linker, connecting N-terminal and M-domains.

### Corresponding large-scale dynamics in Hsp90, Grp94 and HtpG and quasi-rigid domain motions

The insight gained so far into specific common aspects of the chaperones' internal dynamics is here used to introduce and apply a novel comparative analysis scheme that can be aptly used to make manifest the relatedness of the functionally-oriented movements in Hsp90, Grp94 and HtpG.

To the best of our knowledge, the only presently available quantitative method for detecting and exposing correspondences in the internal dynamics of structurally different proteins is the dynamics-based alignment scheme [37], [38], [39], [40], [41], [42], [43]. In this approach, a protein's structural fluctuations or rearrangements are described as a linear superposition of a small number of essential-dynamical spaces obtained, e.g. from the principal component analysis (PCA) of covariance matrices [45]. This strategy would not be appropriate in the present context, because of the very large-scale nature of Hsp90 inter-domain motion. In fact, Hsp90 structures reconstructed by linearly superposing few projected modes would contain unphysical distortions, such as virtual Cα-Cα bonds stretched by up to 6.8 Å.

An alternative and physically appealing approach is suggested by the marked block character of the distance fluctuation matrices in **Figure 4**. This pattern reflects the alternation of regions with small and large fluctuations of inter-residue distances. It therefore indicates that the chaperones' internal dynamics primarily results from the relative motion of a limited number of quasi-rigid domains [42], [43].

**Figure 6:**
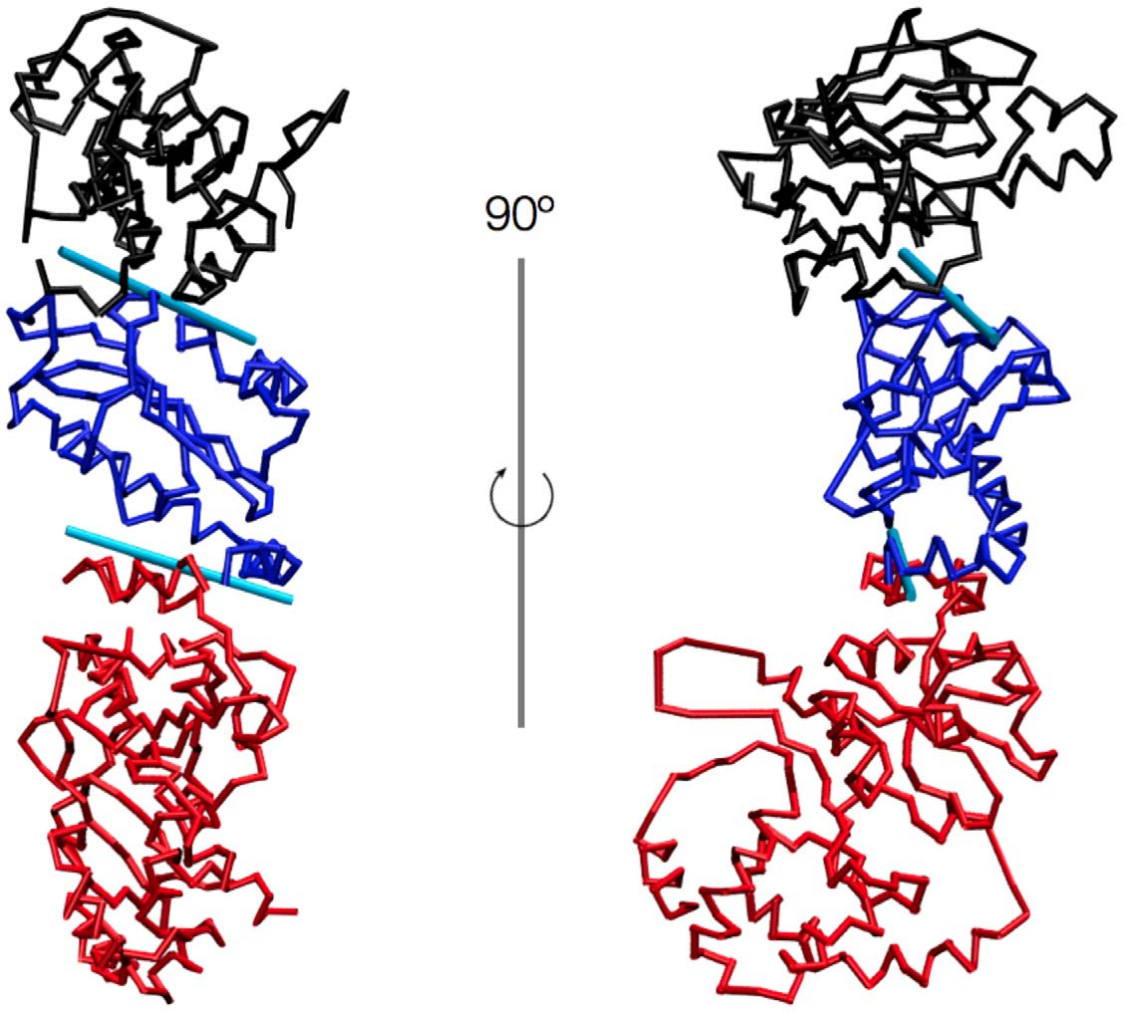
Fitting of the meta-trajectory with only rigid-body movements. Two orthogonal views of the template protomer used to fit all configurations of the Grp94, Hsp90 and HtpG meta-trajectory using only rigid-body movements of the two-terminal quasi rigid domains (black for N-term and red for C-term) respect to the middle (blue) one. The protomer is 525 amino acids long and is the structure closest to the average configuration of the full meta-trajectory. The optimal rotation axes are shown as cyan cylinders. The position and orientation of these axes are identified with an iterative procedure to allow the N-term and the C-term domains to perform those movements which best fit, on average, all frames in the meta-trajectory (see also **Figure 7** for further details). It is worth noting that the axes are not forced a priori to pass through one of the two boundary residues at the interface between two domains.

In order to ascertain the possibility to use a quasi-rigid domain description to capture the protomer motion across all simulations, we proceeded by first identifying a set of residues that can be put in one-to-one correspondence among the proteins. The set, from which a handful of highly mobile exposed residues were removed, consisted of 525 amino acids per protomer (out of 641, 653 and 624 for Hsp90, Grp94 and HtpG, respectively). Next, all the simulated MD trajectories were filtered by retaining only the Cα atoms of the 525 corresponding amino acids and combined into a “meta-trajectory” spanning a total of 1800 ns (900 ns per protomer). Because the “meta-trajectory” combines (reduced) protomeric conformations of the three-chaperones in Apo, ADP-bound and ATP-bound states, its conformational breadth is very large. In fact, individual conformations are at ∼10 Å RMSD from the reference “meta-trajectory” conformation (the one closest to the time-averaged structure) shown in **Figure 6**.

**Figure 7:**
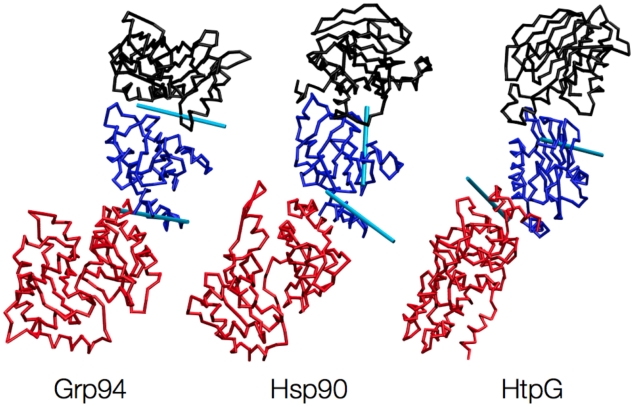
Different views of the optimal rotation axes identified for each part of the meta-trajectory corresponding to a specific chaperone, separately. The template structures have different configurations, each one being the closest to the corresponding chaperone's protomer average structure.

The fluctuations of pairwise Cα distances in the “meta-trajectory” were used to optimally divide the corresponding amino acids into quasi-rigid domains [42], [43]. It was found that as few as three-rigid domains suffice to describe well the conformational space spanned by the “meta-trajectory”. Specifically, 61% of the mean square fluctuation of the meta-trajectory can be accounted for by the relative rigid-body displacements (translations and rotations) of the three quasi-rigid domains shown in **Figure 6**. By inspecting **Figure 6** it is noticed that only the N-terminal quasi-rigid domain matches one of the standard structural subdomains (see also **Figure 7**). The other two quasi-rigid domains are, in fact, separated by the H4–H6 three-helix bundle at the interface between the M-large and M-small structural subdomains [14], [22]. The rigid-body displacement (instantaneous rotations and translations) of these domains can match any configuration in the “meta-trajectory” to an average RMSD of around 3.5 Å; the 81% of the frames are fitted within 4 Å, while the frame having the maximum RMSD does not exceed 5.8 Å (**Figure S10**). These values are remarkably small compared to the typical RMSD differences reported above.

The main corresponding large-scale movements across the three chaperones can be gleaned by restraining the motion of the two terminal quasi-rigid domains to pure rotations around an axis that is fixed with respect to the central domain (see Materials and Methods). **Figure 6** shows the tripartite reference protomer together with the two optimal rotation axes that best approximate the motion of the terminal quasi-rigid domains over the entire “meta-trajectory” (see **Figure 7** for the axes of the individual Grp94, Hsp90 and HtpG trajectories).

The restricted domain motion still allows for capturing ∼55% of the covariance of the original “meta-trajectory”. Any configuration in the Grp94, Hsp90 or HtpG simulations can still be matched to an average RMSD of 7.4 Å, see **Figures 6** **,** **7** **and Video S1, Video S2 and Video S3**. This is a notable result because it is comparable to the resolution typically achieved in cryo-em or SAXS studies of flexible proteins of several hundred amino acids.

The results highlight that the fundamental aspects common to the three chaperones are the rotary motions of the combined M-small+C-terminal macrodomain and the N-terminal domain relative to the M-large one. Notice that the axis of the macrodomain passes through the end of the H4–H6 bundle, which was discussed in a previous section in connection with other ligand-dependent properties. This is noteworthy, because the axis location and orientation was not constrained a priori to lie exactly at the boundary of the quasi-rigid domains.

This fact vividly illustrates that, in spite of the diversities in the initial structural arrangements of the molecules, a common mechanism presides their ligand-dependent large-scale motion. In fact, the hinge located at the interface between the M-large and the M-small domains is highlighted in all proteins as a mechanical hotspot that is capable of modulating the motion of the C-terminal domain in a ligand-dependent manner. It is worth stressing that such key mechanical role is not evident from the standard structural partitions of the protomeric domains.

## Discussion

The function of a large number of proteins and enzymes is determined by the synergic interplay of structure and dynamics, often modulated by substrate binding or covalent modifications. Although many mechanisms have been elucidated [61], [62], [63], [64], [65], [66], [67], the question of how atomic fluctuations are connected to the large-scale functional motions remains a major open problem that is actively investigated experimentally and computationally.

Here, we focus on a specific aspect of this general problem by considering how the large-scale internal dynamics of members of the Hsp90 family is affected by the bound nucleotide. In fact, the conformational turnover of these chaperones depends primarily on ATP binding and hydrolysis (though interaction with co-chaperones and even client proteins is expectedly important for the balance of alternative states in vivo [11], [68]). Characterizing the salient aspects of these chaperones' functional dynamics using atomistic MD simulations has proved challenging. Besides the unavoidable time-span limitations of MD simulations, a key obstacle is the very large-scale character of the chaperones' conformational rearrangements. As a consequence, powerful techniques such as principal component analysis [45], where the internal dynamics is projected on the linear space spanned by a small number of essential dynamical modes [44], cannot be used for a faithful representation of the simulated trajectory.

To overcome these difficulties and to gain insight into the functional dynamical aspects that are common to various members of the Hsp90 family, we introduced a novel multiscale method for analyzing and comparing MD simulations of related, but structurally-different, proteins. Specifically, the method is applied to atomistic MD simulations of the Apo, ADP-bound and ATP-bound states of mammalian Grp94 [16], yeast Hsp90 [14] and *E.coli* HtpG [17]. The molecules differ noticeably for structural organization and are therefore ideal benchmarks for general methods aimed at identifying common, corresponding dynamical traits in the proteins. Indeed, our integrated results allowed to pinpoint common mechanistic properties and to obtain a unified view of the relevant motions linked to function at different levels of resolution.

### Multiscale analysis of Hsp90 chaperones

Our analysis proceeded by comparing the dynamics of Hsp90, Grp94 and HtpG in various ligand-bound states at increasing length-scales: from the atomistic level to the protein domain one.

We started by considering the fluctuations of the distances of all pairs of amino acids and identified the regions experiencing the largest deformation of their local structural neighborhood (strain) [41], [42] upon change of the bound ligand.

Importantly, across all three chaperones, four common regions were identified: the nucleotide binding site in the N-terminal domain (ATP-lid, N-M interface), the aromatic cluster in the H4–H6 helix bundle located in the Middle domain, the core of the C-terminal domain and finally the C-terminal loops defining the allosteric binding pocket for C-terminal inhibitors of Hsp90 [36].

By analyzing the various terms of the proteins' internal energy it is established that the ligand-dependent changes in the protomeric structures correlate with the strength of the electrostatic interaction between the bound nucleotide and the positively charged Arginine in the Middle domain (see **Table 1** **, and** **Figures 2** **, S3**).

The modulation of this attraction affects the catalytic loop (containing Arg448 in Grp94, Arg380 in Hsp90 and Arg336 in HtpG) and, more notably, the cluster of aromatic residues at the end of the H4–H6 three-helix bundle in the Middle domain. In fact, going from the ATP to the ADP state, the increased separation of the nucleotide-binding domain and the catalytic loop in the Middle domain causes the end of the H4–H6 bundle to partially unfold/unpack. This, in turn, leads to the opening of the dimeric clamp. By contrast, the presence of ATP favors the stabilization of the aromatic cluster in an optimally-packed organization.

The fluctuation analysis of the 1800-ns-long “meta-trajectory”, obtained by combining the atomistic trajectories of the single protomers of the three chaperones in all considered ligand-bound states, was next used to subdivide the protomers of the Hsp90, Grp94 and HtpG chaperones into corresponding quasi-rigid domains. The subdivision into quasi-rigid domains lends naturally to describe the large-scale motion of the chaperones with a minimal number of degrees of freedom. In fact, as much as 61% of the “meta-trajectory” mean square fluctuations can be accounted for by the relative rigid-like movements (translations and rotations) of merely 3 quasi-rigid domain (see **Figures 1** **,** **4** **,** **6** **,** **7**). With reference to the structural subdomains, the quasi-rigid (dynamical) ones correspond to: the N-terminal domain, the portion of Middle domain corresponding to the M-large subdomain (including β-sheets S1 to S5 and α-helices H1 to H5) [14], and to the combination of the M-small (comprising H6 to H10) and C-terminal domains (see **Figures 1** **,** **4** **,** **6** **,** **7**).

A minimalistic, though viable, description of the large-scale dynamics that is shared by the three chaperones is obtained by restricting the motion of the two side quasi-rigid domains to consist exclusively of rotations around axes that are fixed (same position and orientation) to the corresponding core of Hsp90, Grp94 and HtpG. By doing so, only two degrees of freedom (the instantaneous rotation angles) are needed to parameterize the “meta-trajectory” and yet it is still possible to account for 55% of its covariance.

The location of the two optimal axes of rotation for the quasi-rigid domains was next used to single out regions that have a crucial mechanical role in the large-scale motion of all three chaperones and hence ought to represent good target sites for inhibiting drugs. Consistently with this observation, one of them is the interface between the N-terminal and Middle domains which corresponds to a previously identified functional site [20], [49], [50], [51]. The second one is centered on the aromatic cluster at the end of the three-helix bundle located at the interface between the large and small sub-portions of the protomeric Middle domain. This latter site was not pointed out before as being functionally relevant and hence could represent a new druggable site for the discovery/design of a novel class of allosteric inhibitors active on the chaperones, thus increasing the chemical space of possible Hsp90 inhibitors for the treatment of different diseases.

### Model for the conformational mechanisms of Hsp90 chaperones

Based on the above results it is possible to formulate a consistent, unified framework for the conformational mechanisms of Hsp90, Grp94 and HtpG.

The primary direct effect ascribable to the specific nucleotides in all three proteins is the modulation of the Coulombic interactions with the positively charged residues in the catalytic loop and in Middle domain (see **Table 1**). These interactions are highest in the presence of ATP, and their attractive character favors the approach of the N-terminal domains through a rotation around the hinge passing at the interfaces with the Middle domains. In fact, ATP-binding to different, more open conformations, here exemplified by the starting structures of Grp94 and HtpG, results in the activated state which can eventually evolve to the final closed compact structure of ATP-bound Hsp90, in which the two N-terminal domains share a large and compact interface. In particular, the importance of the inter-protomer contacts at the N-terminal domains for the efficiency of the Grp94 hydrolysis was demonstrated by the experiments of Dollins and coworkers [16] who reported a reduction of the activity in mutants lacking β-strand1 or β-strand1 plus α-helix 1, which are required for N-terminal dimerization. These closure motions are linked to modulations of the fluctuation and strain patterns that extend through the whole structure, which are strikingly similar across the three proteins, independent of the global 3D organization. In particular, the boundary region between M-small and C-terminal domain increases its mobility, which is compatible with maintaining a significant twist at the C-terminal interface, as observed in the closed activated structure.

Switching from ATP to ADP, the catalytic loop containing Arg448 in Grp94, Arg380 in Hsp90 and Arg336 in HtpG experiences particularly high geometrical strain. The deformation of the contact network of this loop correlates to that of the ordered cluster of aromatic residues at the basis of the H4–H6 three-helix bundle in the Middle domain. The electrostatic attraction of the N-terminal and Middle domains decreases and the protomers relax from the closed state (reference structure Hsp90-ADP), through a rotation that causes a partial unfolding of the basis of the three-helix bundle. This sequence results in an opening of the structures with increased mobility of the Middle domains (Grp94-ADP, HtpG-ADP) and in the collapse of the N-terminal domains over the M-domains, as well as in the population of the “open-compact” conformations described by Agard and coworkers (Grp94-ADP). In this framework, the catalytic loop provides a direct mechanical response to the type of bound nucleotide.

Finally, the release of the nucleotide leads to states with no specific dynamic signatures, which can be correlated to the absence of conformational preferences in the absence of ligand. The release of the ligand induces a strain that favors a reduced twist at the C-terminal interface in all Apo systems, converging to the values typical of HtpG, which can be related to the tendency of the proteins to evolve to fully open structures.

### Model comparison and validation against experimental data

As discussed hereafter, the results of the present multiscale analysis are in accord with several experimental findings and contribute to establishing a comprehensive view of the functional mechanics of these chaperones.

The Neckers group identified a conserved hydrophobic motif in β-strands at the C-terminal boundary of the N-domain, whose mutation abrogated Hsp90 function. Hsp90 mutants lacked chaperone activity in vitro and failed to support yeast viability. Chaperone activity was restored by truncation of the charged linker connecting the N-terminal and Middle domains, consistently with the key “mechanical” role of hydrophobic contacts in the N-M hinge region found in our study [20].

In a different study, Vasko and co-workers identified Sansalvamide A (SanA) as a structurally novel Hsp90 inhibitor. Through the use of pull-down experiments in the presence of the three isolated domains of Hsp90, a construct containing only the N-terminal/Middle domains, and the WT protein, the authors were able to show that SanA binds exclusively to the N-terminal/Middle construct of the protein [50]. Building on this experimental evidence, their studies were extended to synthetic SanA derivatives with different activities [49]. Using a docking-based strategy, they were able to generate structure activity relationships for the small molecules, based on their predicted binding to the N-terminal/Middle interface, as indicated by the pull-downs.

Finally, Matts and coworkers [51] considered different classes of modulators/inhibitors of Hsp90 activity and showed that the action of several inhibitors (whose binding site is still unknown) made the hinge located at the Middle domain three-helix bundle not accessible to trypsin-proteolysis. In particular, in the presence of celastrol, gedunin, or H2-gamendazole the cleavage site Lys398 (together with Arg291) in HtpG (corresponding to Lys508 in Grp94, in close proximity to the location of the hinge located in the Middle domain) is protected from degradation. This suggests that this key site identified by our study is shielded either by direct binding of the drugs or by conformational changes induced through allosteric mechanisms [51].

The three-helix bundle in the Middle domain and the N-M interface hinge have also been shown to be involved in the interaction with the ATPase stimulating co-chaperone Aha1 [22]. Indeed, one Aha1 molecule is found to interact in an asymmetric manner with the Hsp90 dimer: the N-terminal domain of Aha1 engages Hsp90 Middle domain making contacts both with the three-helix bundle, and with the residues at the N-M interface (as seen from NMR perturbation studies), which we found to be crucial for the chaperones' functional mechanics.

In this context, results from the Agard group support the importance of the HtpG region around residue 450 as important in regulating conformational changes. Using small-angle X-ray scattering and EM studies, the authors showed that the conformational equilibrium of HtpG can be shifted with pH, and identified His446, in the immediate proximity of the identified dynamic hotspot, as a key residue that controls this pH-dependent equilibrium. Mutagenesis of His446 was shown to successfully modulate the conformational equilibrium at neutral pH.

With regards to the dynamics of the C-terminal domains, the calculated ATP-induced increase in motion amplitude in the core of the C-terminal domain correlates well with the observed increase of hydrogen-deuterium exchange in the C-terminal core of HtpG upon ATP binding [69]. This supports ligand-dependent changes in flexibility/rigidity in this region, in agreement with our data.

Finally, our model of the ATP-induced transition towards a compact state is consistent with available biochemical and structural data for Grp94. In this context, Dollins and coworkers [16] observed that Glu103Ala and Arg448Ala mutations caused an 85% reduction of the ATPase activity. Our findings about the ligand-responsive dynamics of Glu103 and Arg448 – and particularly their small-amplitude, yet correlated motion in the catalytically-competent ATP state – are consistent with this experimental result.

### Conclusions

The ligand-dependent internal dynamics of three Hsp90 chaperones with markedly different structural organization, mammalian Grp94, yeast Hsp90 and *E.coli* HtpG, was studied by advanced numerical schemes. For each chaperone, the dynamics in the ATP-bound, ADP-bound and Apo states was studied using extensive atomistic molecular dynamics simulations, covering a total time span of 900 ns.

The collected data were analyzed within a novel comparative scheme that is aptly used to single out the salient, common traits of the chaperones' internal dynamics. The proposed analysis scheme has a multiscale character in that it allows to bridge between the ligand-dependent local modifications of the chaperones and the resulting large-scale conformational rearrangements of the chaperones' protomers. Compared to other powerful techniques - such as essential dynamics or principal component - the proposed approach overcomes the structural distortions (e.g. of Cα-Cα virtual bonds) caused by reconstructing the very large amplitude internal dynamics by the linear superposition of very few modes. In fact, it is shown that most of the covariance observed across the 900 ns-long combined trajectories can be captured - with no structural distortion - by using merely two degrees of freedom, which are the angles of rotation of the two side quasi-rigid domains relative to the middle, core one. Based on this result we envisage that the same strategy could be profitably used in cryo-em and SAXS contexts as an effective computational aid for structural modeling.

From this analysis, which is general and hence transferable to other contexts, it emerges that the ligand-dependent modulation of the structural rearrangements in both chaperones is governed by a common underlying mechanism. Specifically, the bound ATP favors the closed protomer conformation by the electrostatic attraction of the N-terminal and Middle domains. The interaction strength decreases significantly going from the ATP-state to the ADP-state and finally to the Apo one. As a result, the relative positioning of the N-terminal and Middle domains is progressively less constrained and favors the open and open-extended conformations, respectively.

The closing/opening rearrangements that are crucial for the chaperones' activity cycle are made possible by the presence of two primary hinge regions at the interface of the quasi-rigid middle domain with the two side ones. These hinges appear to be key players in modulating the protomeric conformations of all Grp94, Hsp90 and HtpG chaperones in response to the type of bound ligand. The first hinge site is located at the interface of the N-terminal and Middle structural domains, while the second is located at the end of the H4–H6 helix-bundle at the boundary between the M-small and the M-large structural subdomains. The first hinge site has already been validated as a potential target for inhibition of the Hsp90 activity [20], [50]. Based on our results and on consistent experimental indications, we therefore suggest that the second hinge region could be a good candidate target for inhibition of the Hsp90 chaperones. Hence, in view of further experimental validation, we propose, as a working hypothesis, that the Middle-domain hinge-region, shared by *all* three chaperones despite their different conformational organization and with a distance of about 40 Å from the nucleotide-binding site, is a possible novel allosteric site. Targeting this site by mutations or small molecules might affect the mechanisms of conformational transition that are crucial for chaperone activity. The strategy to discover putative new inhibitors has been described in [36], where we successfully identified C-terminal targeted drug-like compounds with interesting anticancer activities.

The overall approach we propose may represent a novel and effective means of modulating the functions of different members of the Hsp90 family with drugs that intervene by specifically addressing regions crucial for the functional dynamics of the molecule other than the classically targeted active site.

## Materials and Methods

### Molecular dynamics simulations

The same starting structure was used for the simulations of the ATP-, ADP-, and Apo states of each of the three proteins.

The Grp94 structure used for MD simulations is the full length dimer of the canine protein crystallized by Dollins et al. [16], PDB entry 2O1U.pdb. The crystal sequence starts with Met85. The ATP-lid (residues 166–196), which is disordered in the crystal structure, was modeled according to the crystal structure of the ATP-bound-form of the N-terminal domain, PDB entry 1TC0, chain A. The C-terminal helix, (residues 750–765), also missing in the 2O1U structure, was modeled according to the 2O1T dimer conformation of Middle/C-terminal domains. Two further loops, which are disordered in the full dimer crystal structure (a 4 glycine strand replacing the linker connecting residues 286–330 and the loop 396–407), were modeled using the ModLoop server [70]. The ATP complex of Grp94 was modeled from this structure and according to the crystal information of the 2O1U structure. The Apo and ADP simulations were carried out respectively by removing the ligand and by replacing ATP with ADP, according to the corresponding crystal structure (2O1V). The tetrahedral solvation box has a size of 14×12×14 nm^3^ and contains about 71000 solvent molecules and overall more than 228000 atoms. All simulations and the analysis of the trajectories were performed using the 4.0.3 version of the GROMACS software package [71] using the GROMOS96 force field [72], [73] and the SPC water model [74].

The HtpG structure used for MD simulations is the extended full length dimer modeled starting from the crystal of the bacterial protein by the Agard group [17], PDB entry 2IOP.pdb. The missing loop comprising residues 490–499 was modeled by the ModLoop server [70]. The missing stretch of ATP lid of one protomer was modeled symmetrically by superimposing the coordinates of the opposite protomer, where the lid is fully determined. The Apo and ATP simulations were carried our respectively by removing the ligand and by modeling ATP in the binding site according to crystal data of Hsp90 Nterminal domain complexed to ATP. The tetrahedral solvent box has a size of 10×13×15 nm3 and consists of about 60000 solvent molecules and 190000 atoms.

Following the same protocol used for the Hsp90 simulations, for which we refer to [35], each Grp94 and each HtpG system was first energy relaxed with 2000 steps of steepest descent energy minimization followed by another 2000 steps of conjugate gradient energy minimization, in order to remove possible bad contacts from the initial structures. All systems were first equilibrated by 50 ps of MD runs with position restraints on the protein and ligand to allow relaxation of the solvent molecules. These first equilibration runs were followed by other 50 ps runs without position restraints on the solute. The first 20 ns of each trajectory were not used in the subsequent analysis in order to minimize convergence artifacts. Equilibration of the trajectories was checked by monitoring the equilibration of the RMSD with respect to the initial structure, and of the internal protein energy. Production runs span 100 ns for the ligand bound proteins and 50 ns for the Apo system. The electrostatic term was described by using the particle mesh Ewald algorithm. The LINCS [75] algorithm was used to constrain all bond lengths. For the water molecules the SETTLE algorithm [76] was used. A dielectric permittivity, *ɛ* = 1, and a time step of 2 fs were used. All atoms were given an initial velocity obtained from a Maxwellian distribution at the desired initial temperature of 300K. The density of the system was adjusted performing the first equilibration runs at NPT condition by weak coupling to a bath of constant pressure (P_0_ = 1 bar, coupling time τ_p_ = 0.5 ps) [77]. In all simulations the temperature was maintained close to the intended values by weak coupling to an external temperature bath [77] with a coupling constant of 0.1 ps. The proteins and the rest of the system were coupled separately to the temperature bath.

For Hsp90, the simulation protocol is analogous and is fully described in [35]. The starting structure was downloaded from the Protein Data Bank, file 1CG9.pdb [14]. Production runs for the ATP and ADP cases were extended to 100 ns.

### Matrix of distance fluctuations

For each MD trajectory we computed on the time interval 20–100 ns (the first 20 ns trajectory are removed in order to avoid equilibration artifacts), the matrix of distance fluctuations *A*:
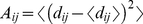
(1)where *d_ij_* is the (time-dependent) distance of the *Cα* atoms of amino acids *i* and *j* and the brackets indicate the time-average over the trajectory. Notice that *A* is invariant under translations and rotations of the molecules and, unlike the covariance matrix, does not depend on the choice of a particular protein reference structure. The *A* matrix, and various quantities derived from it, can be used to characterize the salient elasticity and plasticity properties of a protein undergoing structural fluctuations.

The presence of quasi-rigid domains in the protein of interest should reflect in specific properties of *A*. In fact, pairs of amino acids belonging to the same quasi-rigid domain should be associated with much smaller distance fluctuations than amino acid pairs in different domains [42], [43].

### Hinges of motion

Consistently with the above observation, the hinge regions for the quasi-rigid domain motion can be identified by inspecting the geometric strain profile introduced in ref. [41]. The strain of a given amino acid, *i*, is defined as
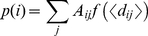
(2)where *j* runs over all protein amino acids, and *f* is a sigmoidal function that restricts the contribution to the sum to amino acids that are within about 5 Å from amino acid *i*: 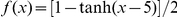, where *x* is expressed in Ångstroms. Hinge regions will be characterized by high values of *p* because in the course of the dynamical evolution their local network of contacts appreciably changes due to the relative motion of neighboring domains.

### Identification of corresponding residues and construction of the “meta-trajectory”

In order to compare the salient features of the internal dynamics of Hsp90, Grp94 and HtpG we first identified the set of their equivalent amino acids. Because of the different length and structural organization of the two chaperones we used to this purpose the FATCAT [78] flexible alignment method.

Specifically, a FATCAT alignment was obtained separately for Hsp90 and HtpG with Grp94; subsequently, we considered the intersection of these two subsets of residues. For each chaperone, we chose as representative protomer the conformation that is closest to the average protomeric structure of the combined Apo, ADP and ATP MD simulations.

The flexible alignment returned 525 corresponding residues (corresponding to ∼82% of each protomer). Next, the individual configurations sampled during each MD trajectory of the proteins were “filtered” by removing the non-corresponding residues. These “reduced” 525-amino acid long configurations in various ligand states were combined into a single “meta-trajectory”. By suitable analysis of this trajectory, it is possible to obtain valuable insight into those aspects of internal dynamics that are common to all chaperones.

### Identification of quasi-rigid domains

The inspection of matrix *A* suggests that the internal dynamics of all three chaperones can be described in terms of relative movements of few quasi-rigid domains connected by hinge regions. The automated identification of a given number of domains was performed, according to the PiSQRD method discussed in refs. [42], [43]. The PiSQRD procedure consists of optimally partitioning the amino acid in a protein (in this case a protomer of one of the three chaperones or of the combined “meta-trajectory”) into a pre-assigned number, *q*, of quasi-rigid units. In a nutshell, this partitioning is identified by minimizing the intra-unit mean-square fluctuations of pairwise distances of amino acids compared to inter-unit pairwise distances. A figure of merit for the viability of the rigid partition is found for each considered number of quasi-rigid domains, *q* = 2,3,4 etc. and based on that the subdivision with the least viable number of domains is retained.

To provide the background for discussing the rotofit procedure described later, we give hereafter a concise account of the PiSQRD algorithm.

Let us suppose to start with a tentative (e.g. random) partitioning of amino acids into *q* putative quasi-rigid domains. Because of the putative quasi-rigid character of the domains, it is expected that (i) the intra-domain structural fluctuations are negligible and (ii) the internal dynamics should mostly consist of relative rotations and translations of the domains.

To ascertain the extent to which these conditions are realized one calculates the quasi-rigid fit “error”
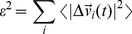
(3)where
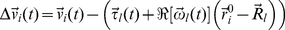
(4)is the difference of the instantaneous velocity of amino acid *i* belonging to the quasi-rigid unit *l* with respect to the pure roto-translational motion that best describes the collective movement of all amino acids in the same domain. In the above expression, 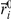 is the positon of the *i*-th amino acid in a reference structure, 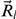 are the coordinates of the domain centre of mass, 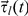 represents the domain translational motion and 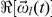 is the rotation matrix describing the instantaneous domain rotation. Clearly the roto-translational parameters, 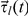 and 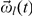 are common to all amino acids belonging to the l-th domain.

The best quasi-rigid domain subdivision is found by minimizing the above error over the tentative assignments of amino acids to the quasi-rigid domains, the latter being explored in a stochastic search in the partition space.

Because of the large-amplitude character of Grp94 and Hsp90 motion the essential dynamical spaces were used only as a first step in our analysis. The returned subdivision was then used as a starting point for minimizing 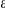, using the complete (and not small-angle linearized approximations) rotation matrices, 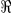.

### Domains rotations

Because the internal dynamics of a protein preserves the chain connectivity, it is intuitively expected that the motion of the *Cα*-trace of a protein should be well described by a purely-rotational motion around a suitable axis passing through one or more *Cα*'s. To identify the dominant rotation axis of a given domain we accordingly enforced that the motion of each amino acid could be well approximated by a pure rigid-body rotation
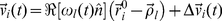
(5)with rotation parameters 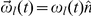, where 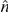 is the unit-norm *time-independent* vector providing the orientation of the rotation axis which is further constrained to pass through the point, 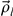. The axes parameters, 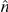 and 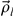, are found by minimizing the internal deformation ε*^2^* as in (3). To make the optimization computationally feasible, the 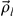
's are restricted to coincide with one of the protein's *Cα* atoms and 500 orientations of 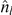 are picked with uniform probability on the unit sphere.

Notice that: (i) the rotation axis does not necessarily pass through the domain center of mass and that (ii) the rotation matrix depends on time only through the instantaneous angle, because the location and directionality of the rotation axes are fixed.

## Supporting Information

Figure S1 **A**, the structural superposition of the C-terminal domains from each protomer of Grp94 (blue), Hsp90 (red) and HtpG (green) shows the different twisting of the interfaces. Subpanel **B** shows the dihedral angle used for the calculation of the twisting of one CTD with respect to the other. Subpanel **C** reports on the time evolution in the different ligand states (running averages black: Apo, green: ADP, yellow: ATP). The color coding for the time evolution of the trajectories is the same as for **A**.
(TIF)

Figure S2 Time evolution of the Solvent Accessible Surface Area calculated at the C-terminal interface in the presence of ATP (black line) and of ADP (red line). **A**, Grp94. **B**, Hsp90. **C**, HtpG. The 20 ns long equilibration stage is indicated with a grey overlay. The interface is defined by residues: 660–675 and 723–735 in Grp94; 591–605 and 651–661 in Hsp90; 552–562 and 602–613 in HtpG.
(TIF)

Figure S3 **A**, Time evolution of the electrostatic interaction energy between the nucleotides and charged residues in the Middle domain of Grp94. **B**, Starting structure (left) and 100 ns snapshot (right) showing the interactions between positively charged residues of the Middle domain and bound ATP at the N-domain of Grp94. Lysines and the putatively catalytic Arginine (Arg448) are shown. **C**, van der Waals interactions between Leu117 and hydrophobic residues of the Middle domain (Val416, Phe417, Ile418).
(TIF)

Figure S4 Representative structures of the most populated clusters of the N-terminal domain in Grp94, highlighting the relative populations and the conformations of the ATP-lid (red tubes).
(TIF)

Figure S5 **A**, Representative structures of the most populated clusters of the N and M-large domains, highlighting the relative orientations of the NTD with respect to the M-domain in the different Grp94 ligand states. **B**, Representative structures of the M-small and C-terminal domains, highlighting the relative orientations of the M-small with respect to the C-terminal domain in the different Grp94 ligand states. In this case, one single cluster accounts for most of the motion. The arrow highlights the contact between M-small and C-terminal domain.
(TIF)

Figure S6 Representative snapshots of the structural evolution in the Hsp90-ADP simulation.
(TIF)

Figure S7 Local unpacking of aromatic residues observed at the Middle domain in the presence of ADP. **A** Left, Grp94 starting conformation. Right, end conformation detail of the three helix bundle, with Phe484 and Phe432 shown as VdW spheres, in the ATP and in the ADP simulation respectively. **B**, same as above, but for Hsp90. Phe364 and Phe421 are shown as VdW spheres. **C**, HtpG. Phe320 and Phe378 are shown as VdW spheres.
(TIF)

Figure S8 Upper plot: Time evolution of the distance between the centers of mass of the two ADP molecules, bound to the N-terminal domains of Grp94, showing the opening of the dimer clamp during the MD trajectory. Lower plot: Time evolution of the distance between the sidechains of Phe484 and Phe432 of each protomer during the same MD trajectory, showing the increased sidechain separation due to the unpacking of the Middle domain aromatic core.
(TIF)

Figure S9 Time evolution of geometrical strain of a Grp94 (**A**), Hsp90 (**B**) and HtpG (**C**) monomer in the presence of ADP. The units are Å^2^.
(TIF)

Figure S10 RMSD between real meta-trajectory protomer configuration and corresponding rigid domain fit as a function of time. RMSD between each protomeric configuration (“frame”) of the meta-trajectory and its best-fit approximation obtained by relative rigid displacement of the domains in the template protomeric structure (see Fig. 6 in main text). In the top panel, the best-fit is found by allowing for instantaneous (i.e. “frame”-dependent) translations and rotations of the quasi-rigid domain. The average RMSD between the “true” protomeric configurations and the rigidly-fitted ones is equal to 2.3 Å (N-terminal) and 2.5 Å (C-terminal) for Grp94, 3.2 Å (N-terminal) and 4.0 Å (C-terminal) for Hsp90 and 2.8 Å (N-terminal) and 4.7 Å (C-terminal) for HtpG. In the lower panel the best-fit allows only for rotations of the side domains around optimally chosen axes that are fixed (i.e. “frame”-independent) in position and orientation relative to the middle quasi-rigid domain. The maximum RMSD between the meta-trajectories configurations and the rigidly-rotating-domains ones is 5.2, 9.3 and 7.6 Å for Grp94, Hsp90 and HtpG, respectively.
(TIF)

Text S1 Coordinate file for the final structure of the Grp94-ADP simulation.
(TXT)

Text S2 Coordinate file for the final structure of the Grp94-ATP simulation.
(TXT)

Text S3 Coordinate file for the final structure of the Hsp90-ADP simulation.
(TXT)

Text S4 Coordinate file for the final structure of the Hsp90-ATP simulation.
(TXT)

Text S5 Coordinate file for the final structure of the HtpG-ADP simulation.
(TXT)

Text S6 Coordinate file for the final structure of the HtpG-ATP simulation.
(TXT)

Video S1 Solid backbone: Grp94 meta-trajectory. The meta-trajectory consists of all protomeric snapshots (restricted to the 525 amino acids alignable with Hsp90 and HtpG) of Grp94 in the Apo, ADP- and ATP-bound states. The faint backbone represents the best fit to the Grp94 protomers obtained by suitably rotating the two terminal quasi-rigid domains of protomeric template (see **Figure 6**) around the axes shown as cyan cylinders.
(MPG)

Video S2 Solid backbone: Hsp90 meta-trajectory. The meta-trajectory consists of all protomeric snapshots (restricted to the 525 amino acids alignable with Grp94 and HtpG) of Hsp90 in the Apo, ADP- and ATP-bound states. The faint backbone represents the best fit to the Grp94 protomers obtained by suitably rotating the two terminal quasi-rigid domains of protomeric template (see **Figure 6**) around the axes shown as cyan cylinders.
(MPG)

Video S3 Solid backbone: HtpG meta-trajectory. The meta-trajectory consists of all protomeric snapshots (restricted to the 525 amino acids alignable with Grp94 and Hsp90) of HtpG in the Apo, ADP- and ATP-bound states. The faint backbone represents the best fit to the Grp94 protomers obtained by suitably rotating the two terminal quasi-rigid domains of protomeric template (see **Figure 6**) around the axes shown as cyan cylinders.
(MPG)
