# Supplementary figures and images for: Corresponding Functional Dynamics across the Hsp90 Chaperone Family: Insights from a Multiscale Analysis of MD Simulations
Source: PLoS Comput Biol. 2012 Mar 22;8(3):e1002433. doi: 10.1371/journal.pcbi.1002433 (PMC3310708; doi:10.1371/journal.pcbi.1002433)

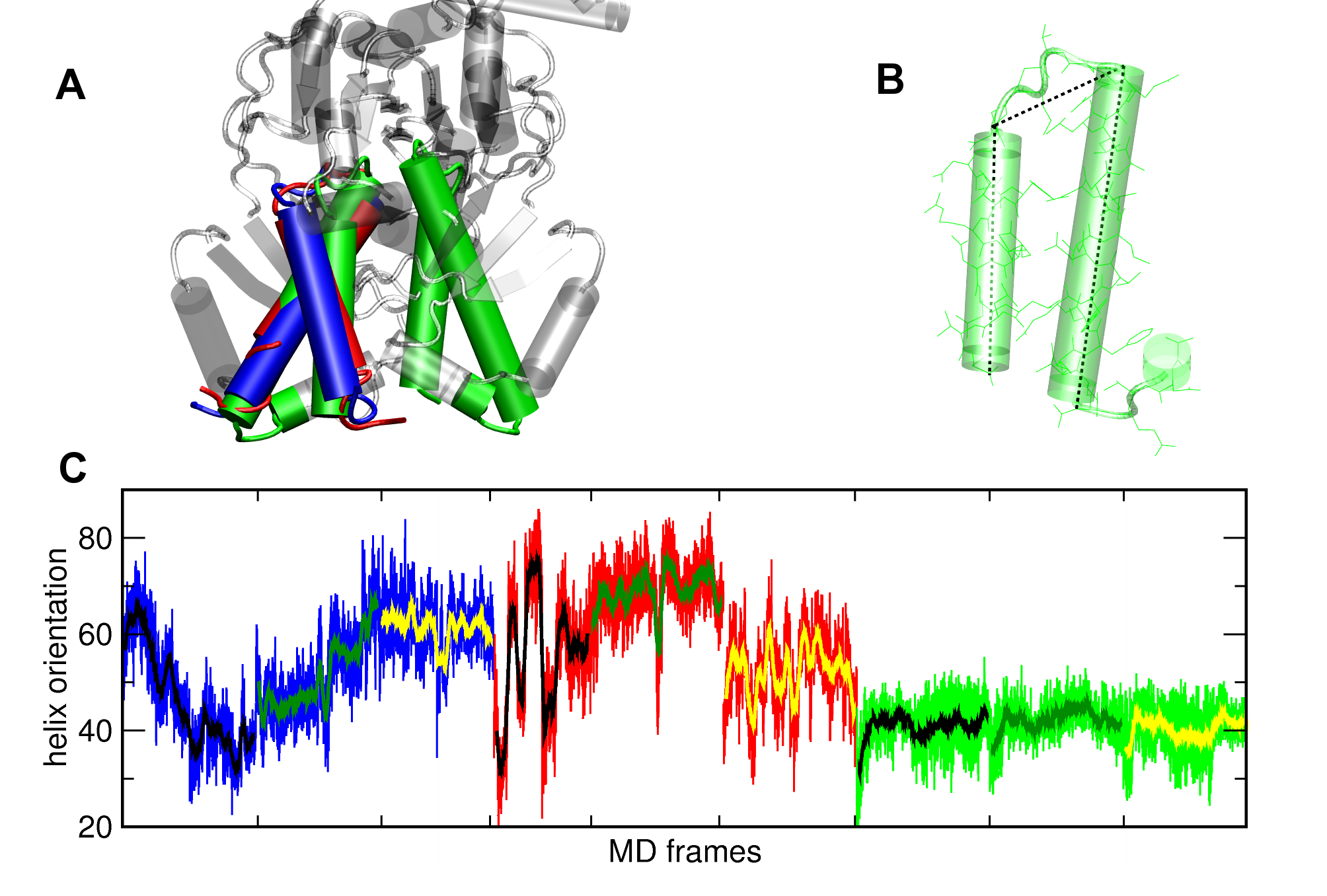

Supplement: Figure S1 — A, the structural superposition of the C-terminal domains from each protomer of Grp94 (blue), Hsp90 (red) and HtpG (green) shows the different twisting of the interfaces. Subpanel B shows the dihedral angle used for the calculation of the twisting of one CTD with respect to the other. Subpanel C reports on the time evolution in the different ligand states (running averages black: Apo, green: ADP, yellow: ATP). The color coding for the time evolution of the trajectories is the same as for A. (TIF) [file pcbi.1002433.s001.tif]

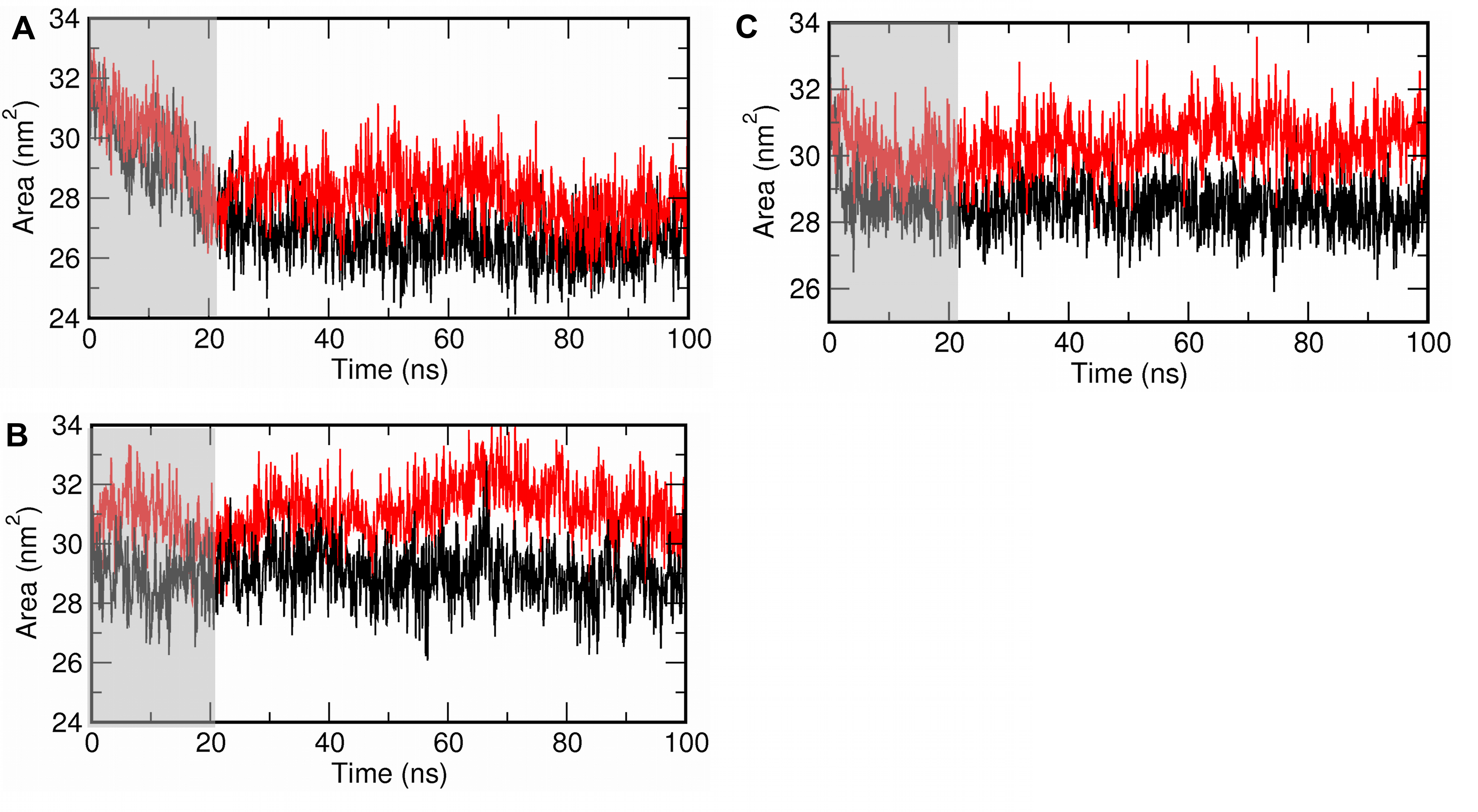

Supplement: Figure S2 — Time evolution of the Solvent Accessible Surface Area calculated at the C-terminal interface in the presence of ATP (black line) and of ADP (red line). A, Grp94. B, Hsp90. C, HtpG. The 20 ns long equilibration stage is indicated with a grey overlay. The interface is defined by residues: 660–675 and 723–735 in Grp94; 591–605 and 651–661 in Hsp90; 552–562 and 602–613 in HtpG. (TIF) [file pcbi.1002433.s002.tif]

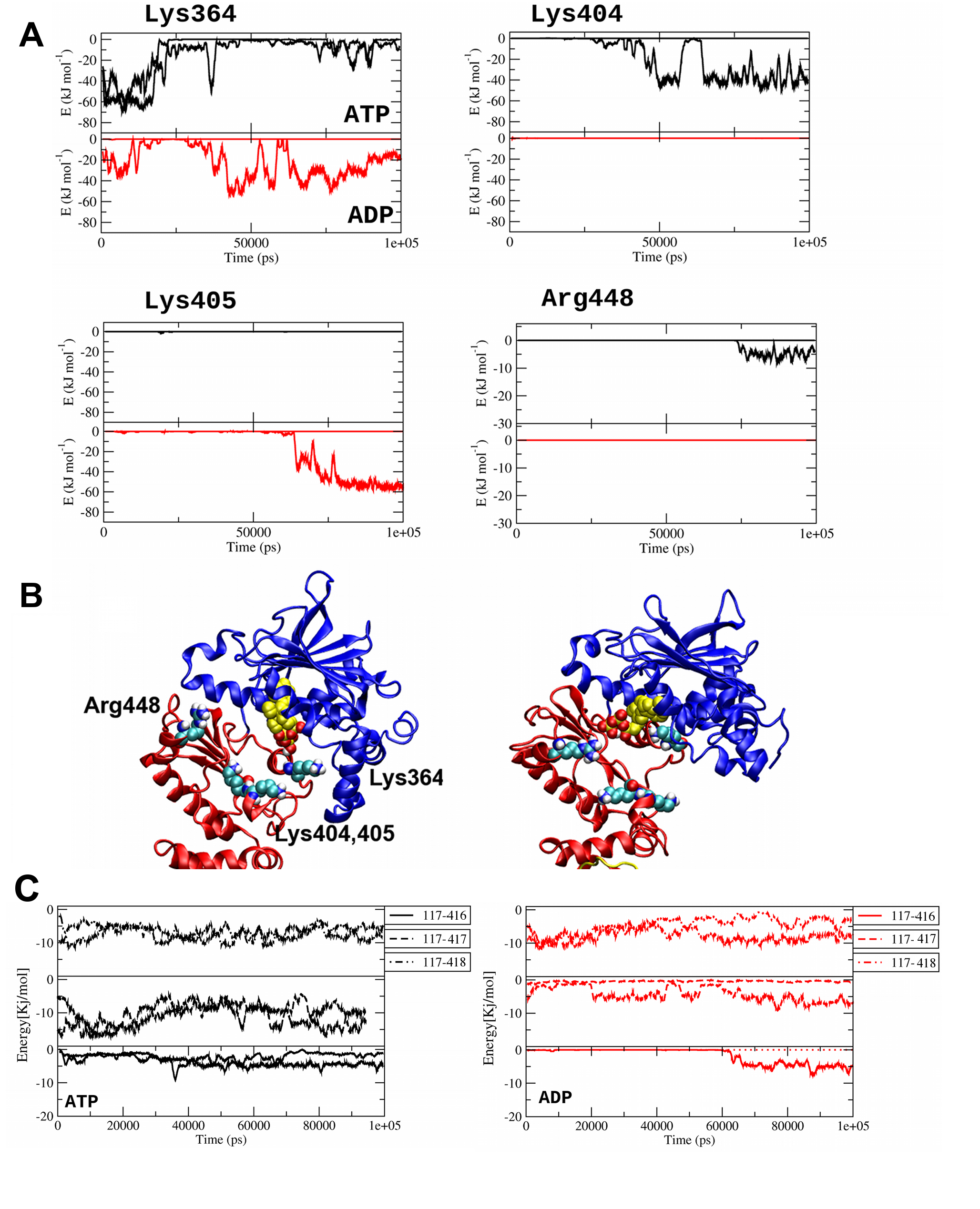

Supplement: Figure S3 — A, Time evolution of the electrostatic interaction energy between the nucleotides and charged residues in the Middle domain of Grp94. B, Starting structure (left) and 100 ns snapshot (right) showing the interactions between positively charged residues of the Middle domain and bound ATP at the N-domain of Grp94. Lysines and the putatively catalytic Arginine (Arg448) are shown. C, van der Waals interactions between Leu117 and hydrophobic residues of the Middle domain (Val416, Phe417, Ile418). (TIF) [file pcbi.1002433.s003.tif]

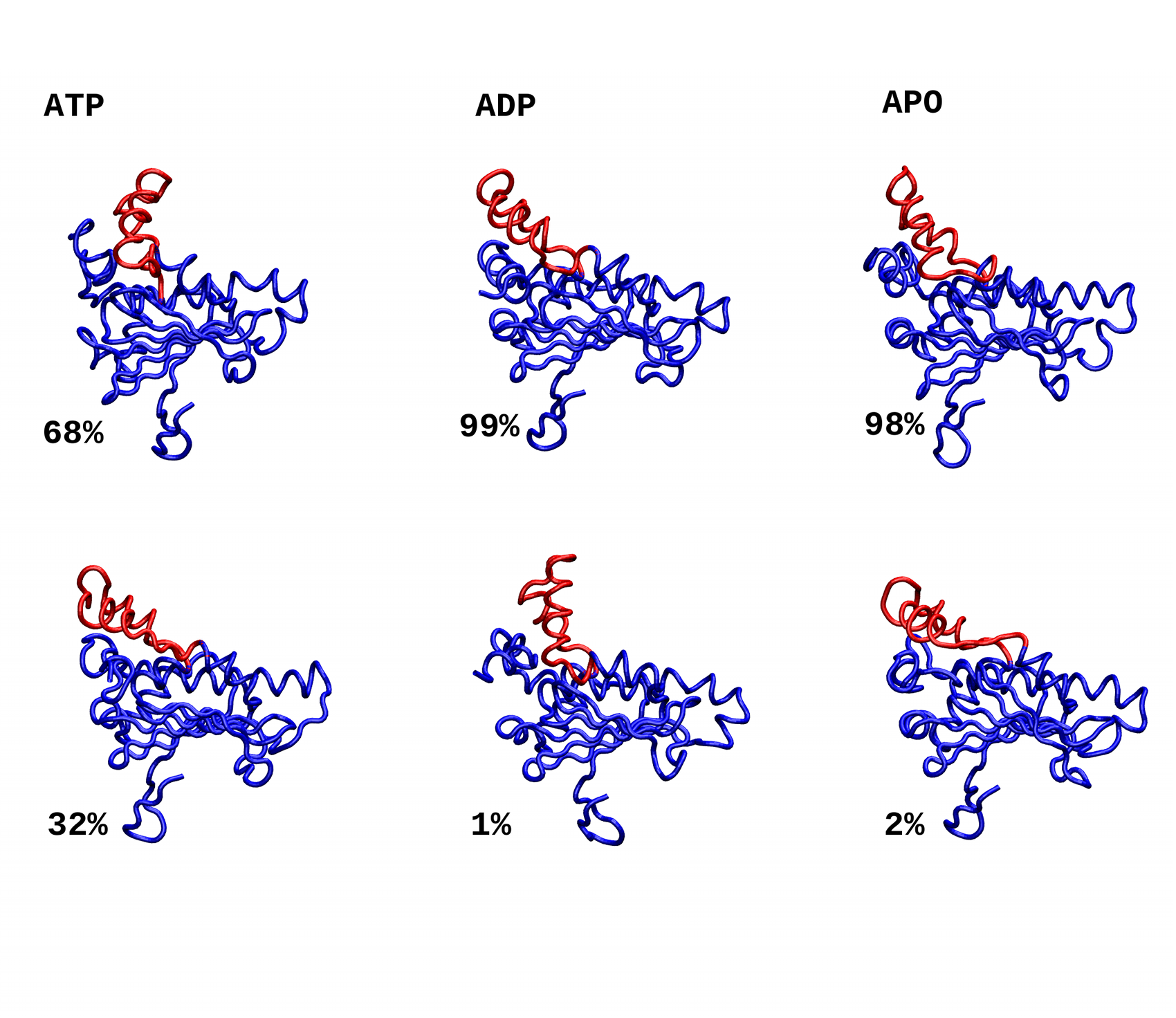

Supplement: Figure S4 — Representative structures of the most populated clusters of the N-terminal domain in Grp94, highlighting the relative populations and the conformations of the ATP-lid (red tubes). (TIF) [file pcbi.1002433.s004.tif]

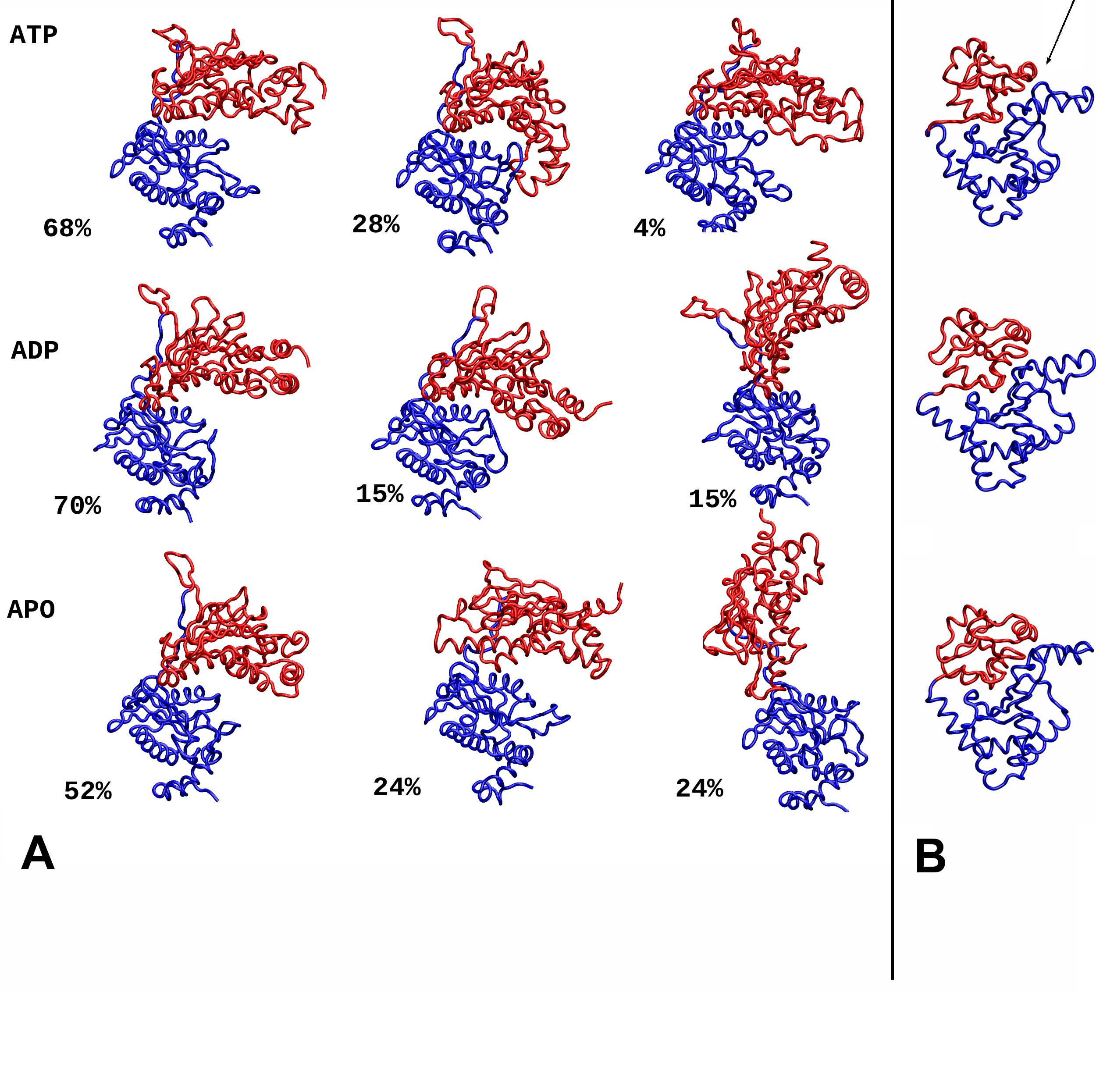

Supplement: Figure S5 — A, Representative structures of the most populated clusters of the N and M-large domains, highlighting the relative orientations of the NTD with respect to the M-domain in the different Grp94 ligand states. B, Representative structures of the M-small and C-terminal domains, highlighting the relative orientations of the M-small with respect to the C-terminal domain in the different Grp94 ligand states. In this case, one single cluster accounts for most of the motion. The arrow highlights the contact between M-small and C-terminal domain. (TIF) [file pcbi.1002433.s005.tif]

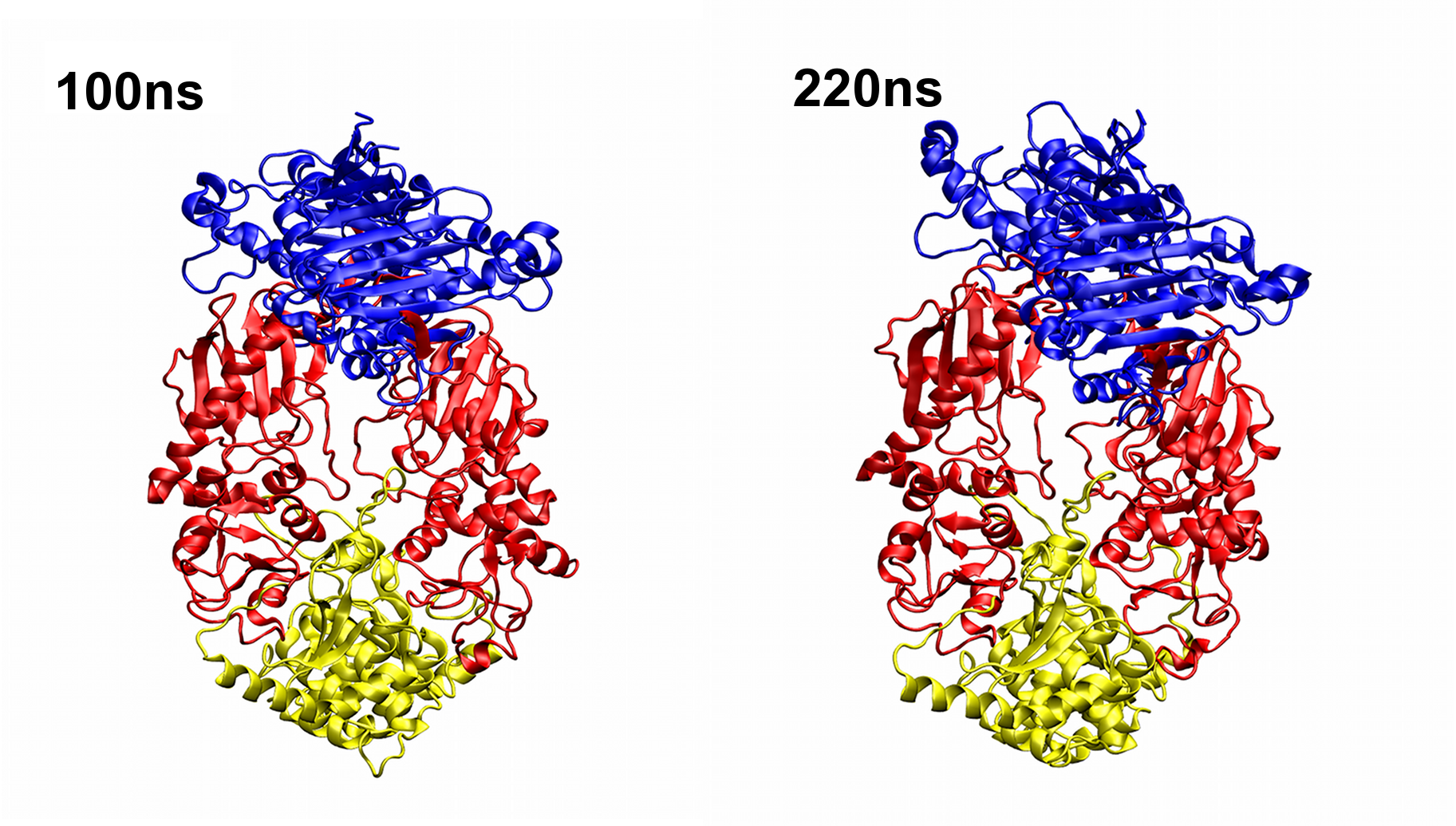

Supplement: Figure S6 — Representative snapshots of the structural evolution in the Hsp90-ADP simulation. (TIF) [file pcbi.1002433.s006.tif]

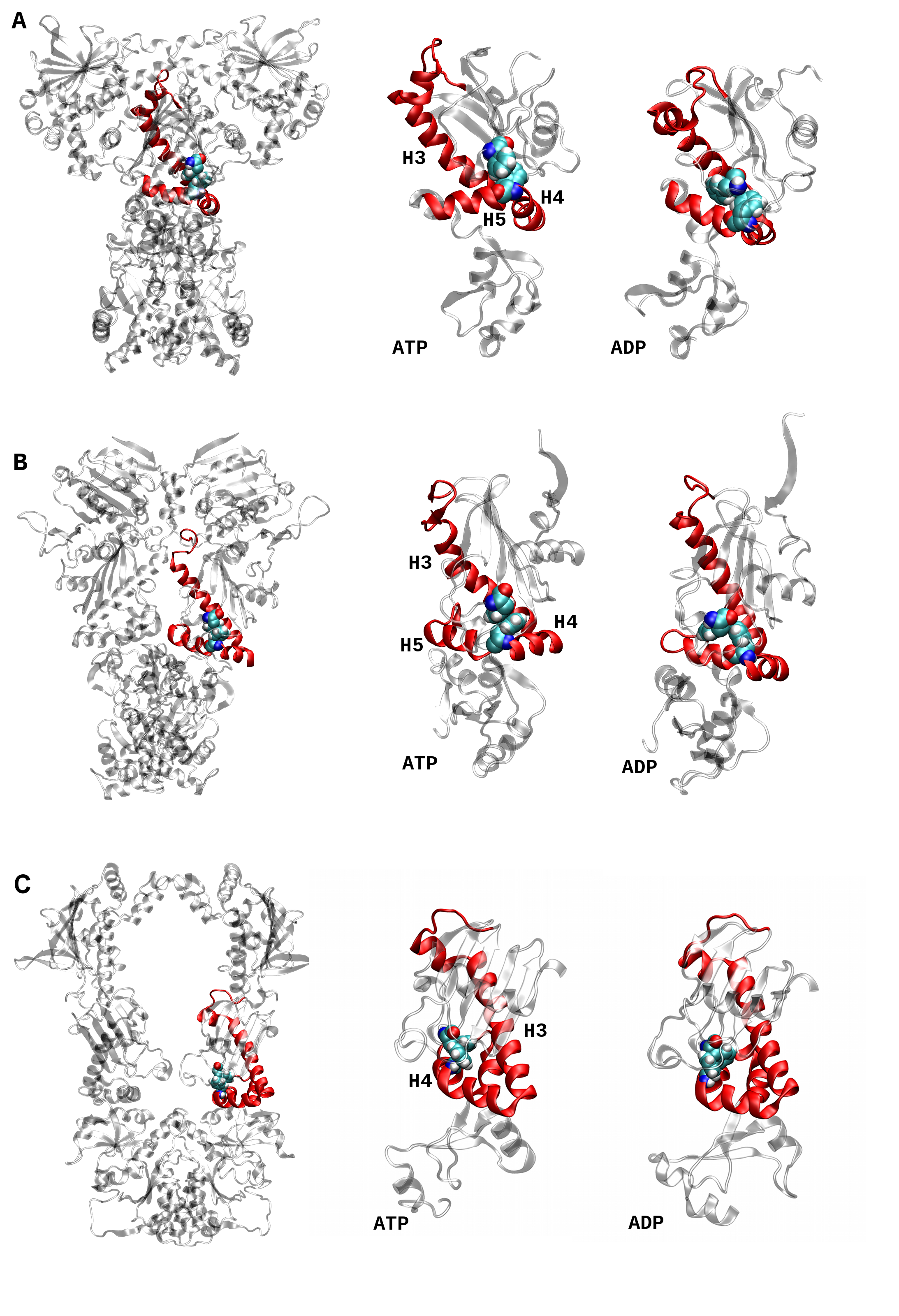

Supplement: Figure S7 — Local unpacking of aromatic residues observed at the Middle domain in the presence of ADP. A Left, Grp94 starting conformation. Right, end conformation detail of the three helix bundle, with Phe484 and Phe432 shown as VdW spheres, in the ATP and in the ADP simulation respectively. B, same as above, but for Hsp90. Phe364 and Phe421 are shown as VdW spheres. C, HtpG. Phe320 and Phe378 are shown as VdW spheres. (TIF) [file pcbi.1002433.s007.tif]

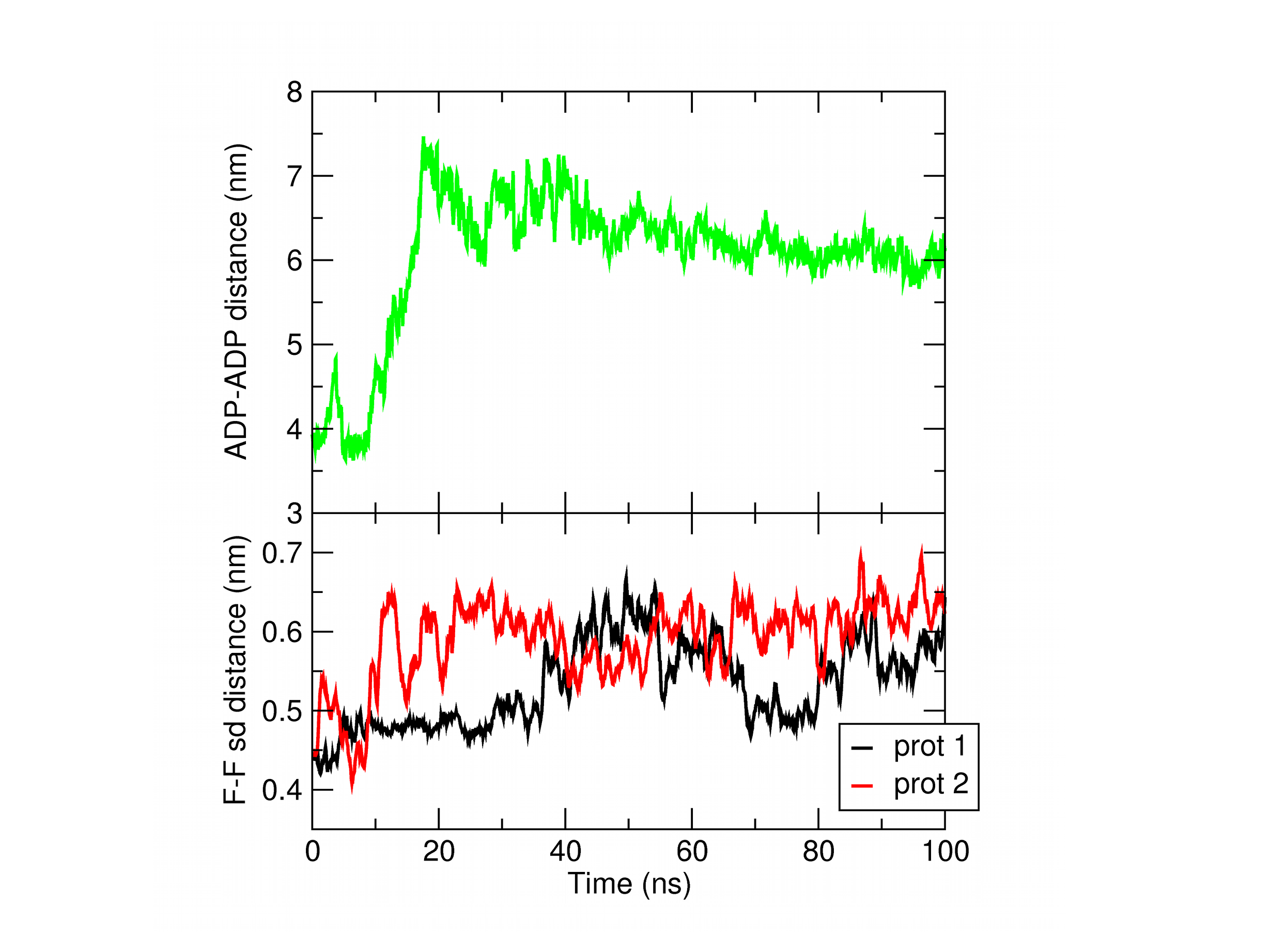

Supplement: Figure S8 — Upper plot: Time evolution of the distance between the centers of mass of the two ADP molecules, bound to the N-terminal domains of Grp94, showing the opening of the dimer clamp during the MD trajectory. Lower plot: Time evolution of the distance between the sidechains of Phe484 and Phe432 of each protomer during the same MD trajectory, showing the increased sidechain separation due to the unpacking of the Middle domain aromatic core. (TIF) [file pcbi.1002433.s008.tif]

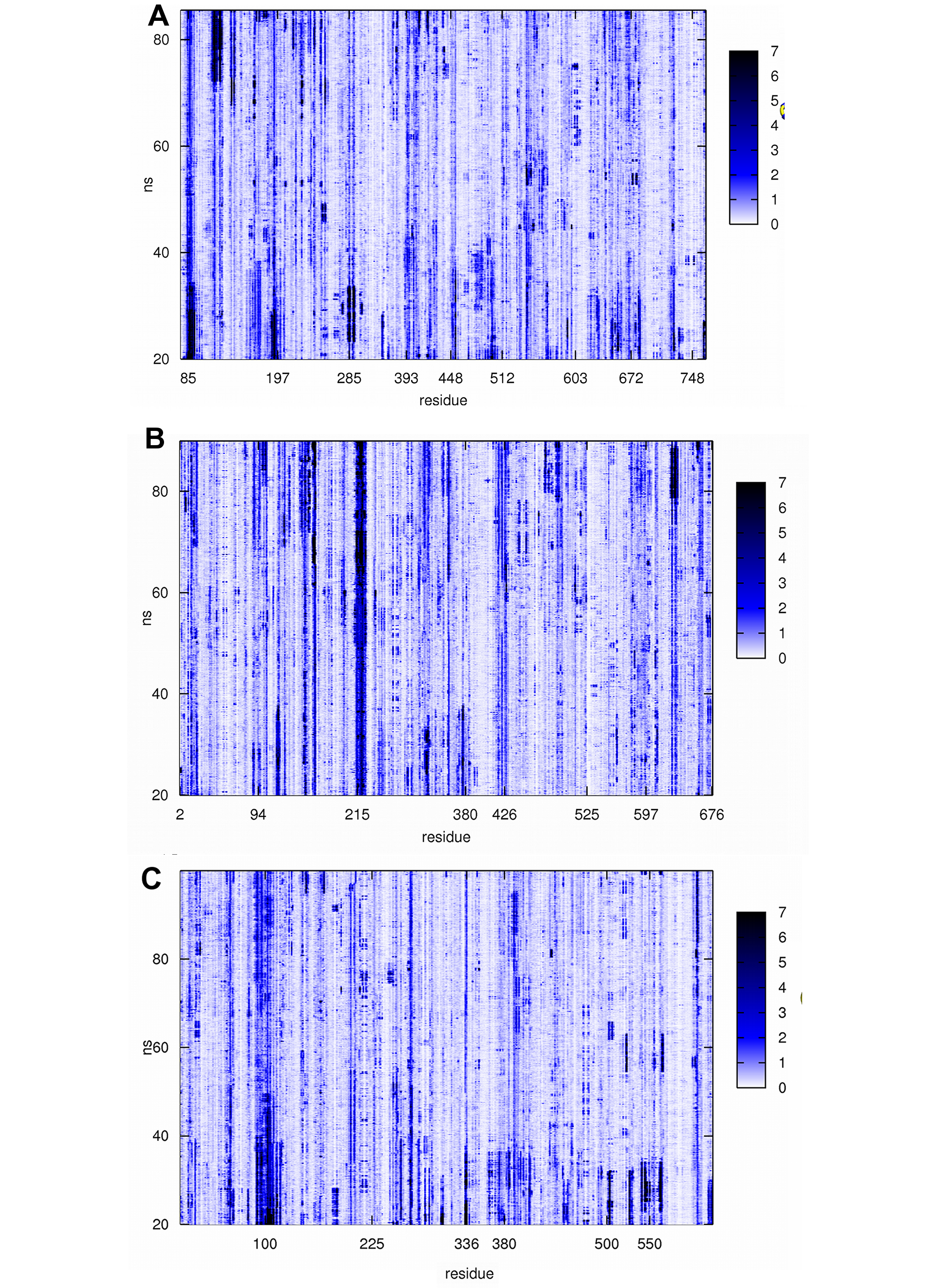

Supplement: Figure S9 — Time evolution of geometrical strain of a Grp94 (A), Hsp90 (B) and HtpG (C) monomer in the presence of ADP. The units are Å2. (TIF) [file pcbi.1002433.s009.tif]

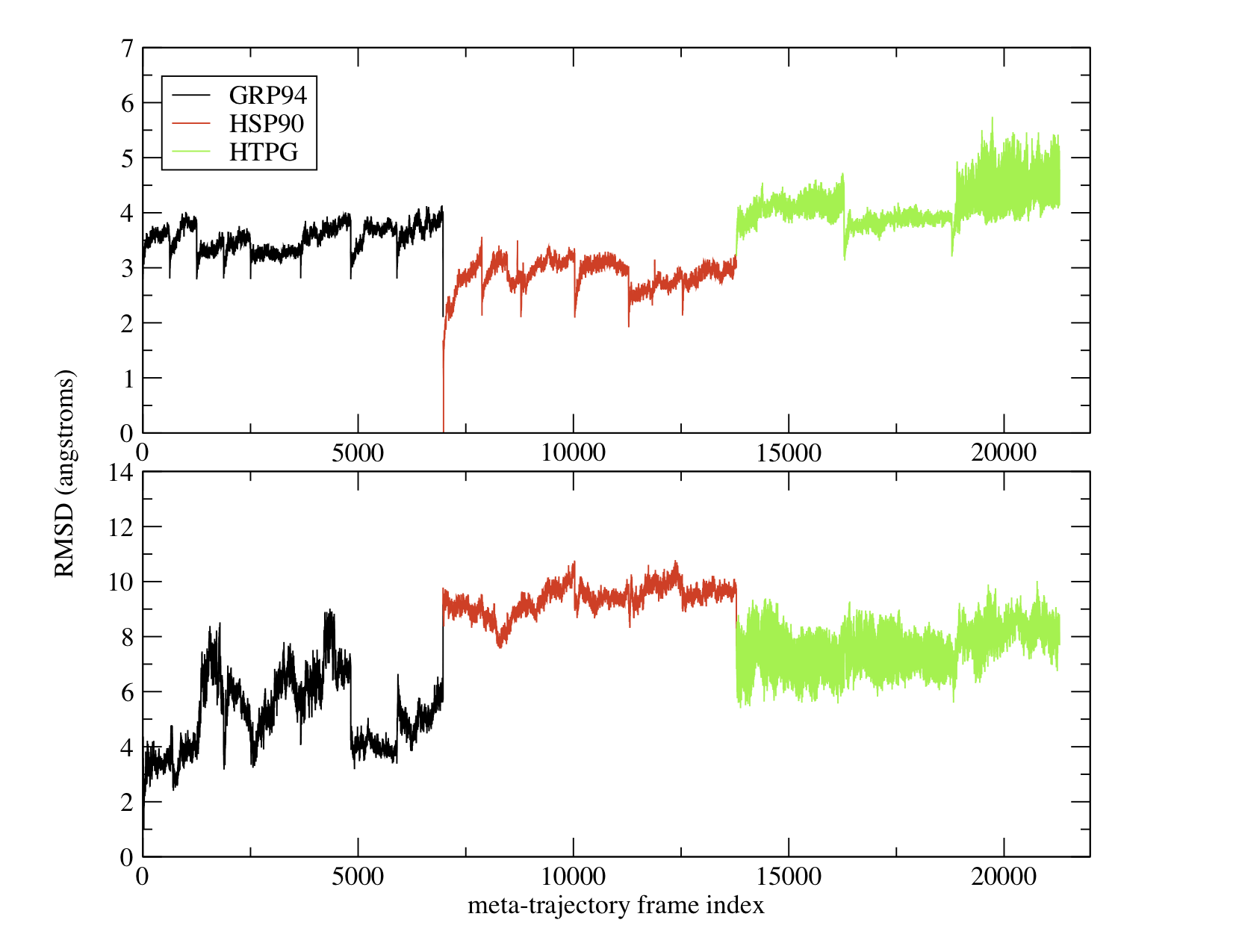

Supplement: Figure S10 — RMSD between real meta-trajectory protomer configuration and corresponding rigid domain fit as a function of time. RMSD between each protomeric configuration (“frame”) of the meta-trajectory and its best-fit approximation obtained by relative rigid displacement of the domains in the template protomeric structure (see Fig. 6 in main text). In the top panel, the best-fit is found by allowing for instantaneous (i.e. “frame”-dependent) translations and rotations of the quasi-rigid domain. The average RMSD between the “true” protomeric configurations and the rigidly-fitted ones is equal to 2.3 Å (N-terminal) and 2.5 Å (C-terminal) for Grp94, 3.2 Å (N-terminal) and 4.0 Å (C-terminal) for Hsp90 and 2.8 Å (N-terminal) and 4.7 Å (C-terminal) for HtpG. In the lower panel the best-fit allows only for rotations of the side domains around optimally chosen axes that are fixed (i.e. “frame”-independent) in position and orientation relative to the middle quasi-rigid domain. The maximum RMSD between the meta-trajectories configurations and the rigidly-rotating-domains ones is 5.2, 9.3 and 7.6 Å for Grp94, Hsp90 and HtpG, respectively. (TIF) [file pcbi.1002433.s010.tif]
